# Charge-State Dependence of Proton Uptake in Polyoxovanadate-alkoxide Clusters

**DOI:** 10.1021/acs.inorgchem.1c02937

**Published:** 2022-03-16

**Authors:** Eric Schreiber, William W. Brennessel, Ellen M. Matson

**Affiliations:** Department of Chemistry, University of Rochester, Rochester, New York 14627, United States

## Abstract

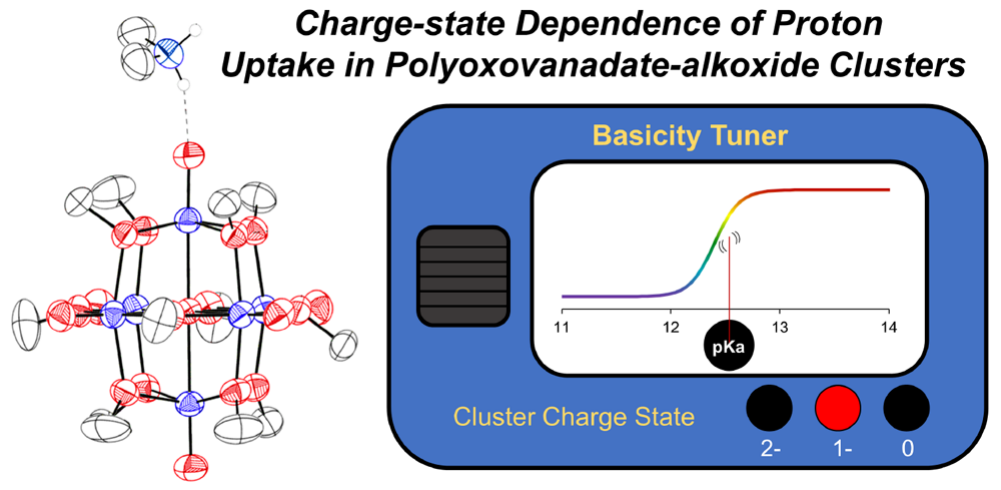

Here, we present an investigation of the thermochemistry of proton uptake in acetonitrile across three charge states of a polyoxovanadate-alkoxide (POV-alkoxide) cluster, [V_6_O_7_(OMe)_12_]^*n*^ (*n* = 2–, 1–, and 0). The vanadium oxide assembly studied features bridging sites saturated by methoxide ligands, isolating protonation to terminal vanadyl moieties. Exposure of [V_6_O_7_(OMe)_12_]^*n*^ to organic acids of appropriate strength results in the protonation of a terminal V=O bond, generating the transient hydroxide-substituted POV-alkoxide cluster [V_6_O_6_(OH)(OMe)_12_]^*n*+1^. Evidence for this intermediate proved elusive in our initial report, but here we present the isolation of a divalent anionic cluster that features hydrogen bonding to dimethylammonium at the terminal oxo site. Degradation of the protonated species results in the formation of equimolar quantities of one-electron-oxidized and oxygen-atom-efficient complexes, [V_6_O_7_(OMe)_12_]^*n*+1^ and [V_6_O_6_(OMe)_12_]^*n*+1^. While analogous reactivity was observed across the three charge states of the cluster, a dependence on the acid strength was observed, suggesting that the oxidation state of the vanadium oxide assembly influences the basicity of the cluster surface. Spectroscopic investigations reveal sigmoidal relationships between the acid strength and cluster conversion across the redox series, allowing for determination of the proton affinity of the surface of the cluster in all three charge states. The fully reduced cluster is found to be the most basic, with higher oxidation states of the assembly possessing substantially reduced proton affinities (∼7 p*K*_a_ units per electron). These results further our understanding of the site-specific reactivity of *terminal* M=O bonds with protons in an organic solvent, revealing design criteria for engineering functional surfaces of metal oxide materials of relevance to energy storage and conversion.

## Abstract 2

Experimental determination of the surface basicity of polyoxovanadate-alkoxide clusters across three oxidation states reveals a charge-state dependence of proton uptake in molecular, organofunctionalized vanadium oxide assemblies.

## Introduction

The interactions of cations with the surface of reducible metal oxides (RMOs) is an important area of research, with implications in the advancement of energy storage technologies^1,2^ and the development of catalysts.^3,4^ In reports on cation uptake into bulk and nanocrystalline materials, analytical techniques such as X-ray diffraction, electrochemistry, and a host of spectroscopies have been used to describe how the bulk structure of a metal oxide is altered upon uptake of cationic species, as well as ascertain the mechanism of cation insertion.^5−9^ Although current analytical techniques provide critical information on global material properties, they do not provide atomically precise detail, for which researchers turn to computational modeling.^5,7,9−11^ To empirically obtain atomic-level insight into these types of interactions, alternatively researchers can turn to the study of molecular model complexes.

Polyoxometalates (POMs) are molecular assemblies composed of multiple redox-active transition-metal oxyanions linked together by bridging oxide units to form three-dimensional structures. The peripheral morphology of POMs resembles the surface of RMOs, with alternating terminal and bridging oxygen atoms composing the surface of the assembly.^12−16^ However, unlike bulk solids, POMs exhibit solubility in organic and aqueous solution, rendering these clusters amenable to analysis using analytical techniques reserved for homogeneous systems. Given the relevance of POMs as homogeneous models for bulk systems, there has been great interest in understanding the role of cations in mediating the electrochemical properties of these assemblies.^17−25^ Charge-compensating interactions between the surface of POMs and protons have been reported to result in the coalescence of individual one-electron-oxidized (1e^–^) redox events into multielectron–multiproton processes located at potentials anodically shifted from those reported for the parent complex.^18−20,22,24,25^ Of note, the affinity of the POM surface for protons has been described as dependent on the degree of reduction of the cluster, with more reduced variants of the metal oxide assembly possessing acid association equilibrium constants with an order of magnitude higher than that of their oxidized counterparts.^18−22,25^ Theoretical and experimental studies have demonstrated that the interactions of protons with the surface of the POM occur at nucleophilic, bridging oxido (μ_2_-O^2–^) sites following reduction of the parent ion.^24,26−28^

The reactivity of terminal oxo moieties in POMs toward protons, on the other hand, has not been as prominently observed. Besides our recent report on an ethoxide-bridged polyoxovanadate cluster (*vide infra*), only one example of the formal protonation of terminal oxido groups of a POM has been reported to date.^29−32^ In a series of papers, the six-electron reduction of a Keggin-type polyoxotungstate, [XW_12_O_60_]^*y*−^ (X = Si, B, H_2_), in an acidic medium was shown to produce a charge-separated complex featuring three "W^IV^O_5_(OH_2_)" sites on a single cluster face. The surface-bound aquo ligands were shown to be labile; refluxing the reduced species in organic solvent results in dissociation of the water moiety from the W^IV^ center, exposing three oxygen-atom vacancies. The cluster is subsequently able to perform deoxygenation of dimethyl and diphenyl sulfoxide, triphenylarsine oxide, and nitrosobenzene.^32^ Whereas this work has intriguing potential implications in catalysis and materials chemistry, very little progress has been made in the formation of oxygen-atom defects via the protonation of other reduced POMs.

Our research group has been investigating the synthesis and reactivity of a family of organofunctionalized polyoxovanadate clusters first reported by Hartl and co-workers.^33^ This subset of POMs is unique because they possess reduced metal centers and low overall charge. The hexavanadate assembly features 12 bridging alkoxide ligands that flank the surface of the Lindqvist core that stabilize reduced variants of the cluster and saturate nucleophilic μ_2_-O^2–^ sites at the surface of the assembly. This, in turn, inhibits these moieties from participating in strong interactions with cations, providing opportunities to isolate and explore the reactivity of *terminal* oxido ligands.

**Scheme 1:**
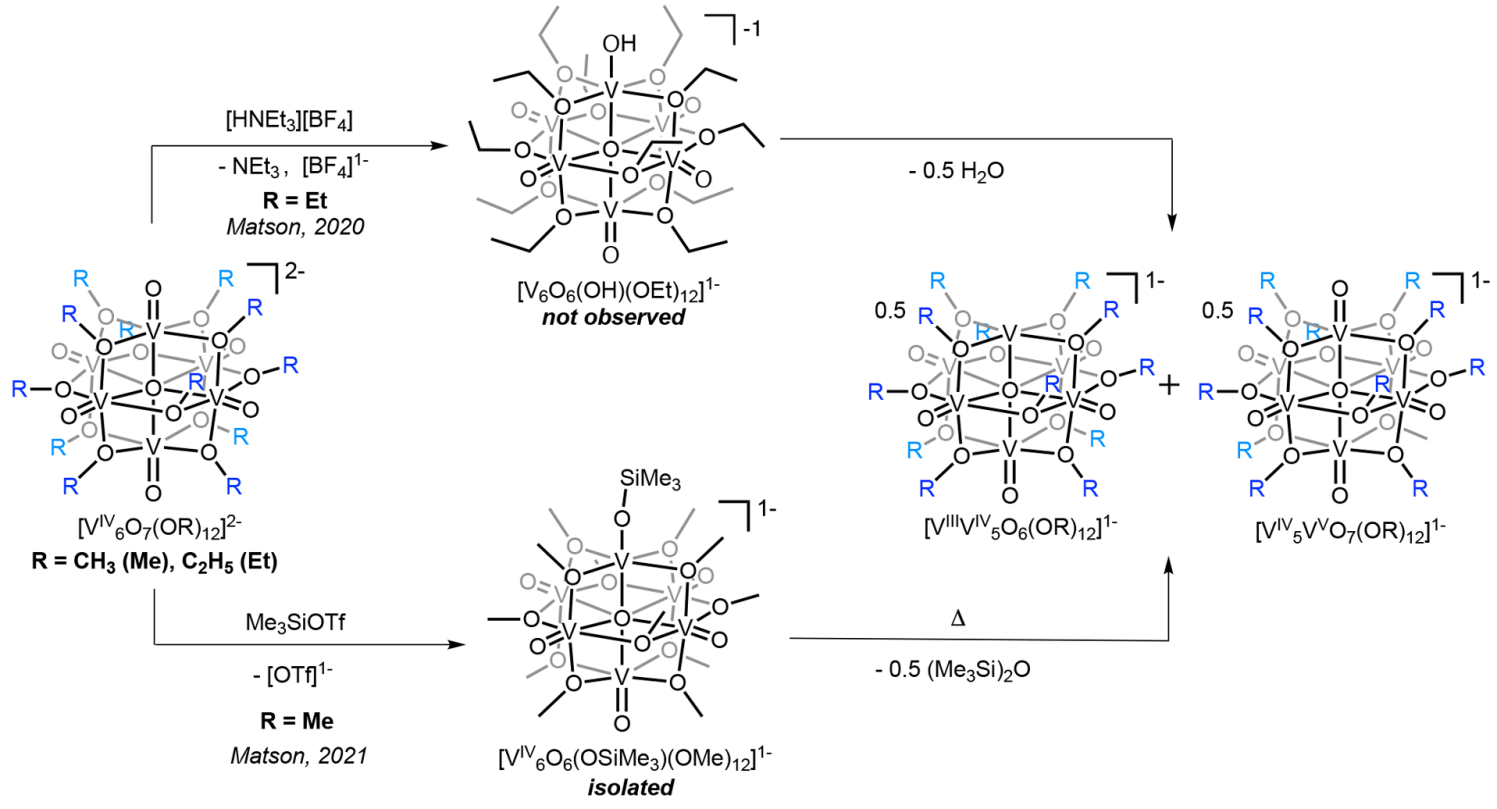
Previous Work Focused on Investigating the Reactivity of Protons with Reduced POV-alkoxide Clusters: Acid-Driven Oxygen-Atom-Vacancy Formation via the Reaction of [V^IV^_6_O_7_(OEt)_12_]^2–^ with [HNEt_3_][BF_4_] [p*K*_a_(MeCN) = 18.82; Top]^34^ and Activation of the Surface Vanadyl Moiety of **1-V**^**IV**^_**6**_**O**_**7**_^**2–**^ with TMSOTf Revealing the Isolation of a Siloxide-Functionalized POV-alkoxide Cluster (Bottom)^35^

To this end, we recently reported the reactivity of the reduced polyoxovanadate-alkoxide (POV-alkoxide) cluster [V^IV^_6_O_7_(OEt)_12_]^2–^ with an organic acid.^34^ The addition of 1 equiv of triethylammonium tetrafluoroborate ([HNEt_3_][BF_4_]) to [V^IV^_6_O_7_(OEt)_12_]^2–^ results in the formation of a 1:1 mixture of a cluster with an oxygen-atom vacancy, [V^III^V^IV^_5_O_6_(OEt)_12_]^1−^, and the 1e^–^ species [V^IV^_5_V^V^O_7_(OEt)_12_]^1−^. In our original report, we include a proposed mechanism for the aforementioned reaction: protonation of a terminal V^IV^=O moiety at the surface of the cluster is believed to result in the formation of a transient, hydroxide-substituted POV-alkoxide (e.g., [V_6_O_6_(OH)(OEt)_12_]^1−^), which subsequently disproportionates via proton-coupled electron transfer (PCET) to form [V^III^V^IV^_5_O_6_(OEt)_12_]^1−^ and [V^IV^_5_V^V^O_7_(OEt)_12_]^1−^ (Scheme 1). Further evidence in support of this mechanism was disclosed in our recent publication describing the reactivity of a silylium ion with the reduced vanadium oxide assembly;^35^ here, it is important to note that silylium ions have been described ubiquitously as bulky surrogates for protons.^36−40^ The addition of 1 equiv of trimethylsilyl trifluoromethylsulfonate (TMSOTf) to [V^IV^_6_O_7_(OMe)_12_]^2–^ (**1-V**^**IV**^_**6**_**O**_**7**_^**2–**^) results in formation of the siloxide-functionalized cluster [V^IV^_6_O_6_(OSiMe_3_)(OMe)_12_]^1−^ in excellent yield, strengthening our claim that the addition of organic acids results in protonation of a terminal oxido ligand at the surface of the assembly.

On the basis of the proposed mechanism for proton-induced oxygen-atom-defect formation at the surface of POV-alkoxide, the p*K*_a_ of the transient hydroxide-substituted assembly [V_6_O_6_(OH)(OMe)_12_]^1–^ is envisaged as an important thermochemical parameter in defining the reactivity of the cluster with organic acids. As such, this work targets elucidation of the basicity of terminal oxido moieties in POM architectures to establish trends for activation of these distinct surface sites in metal oxide assemblies. Here, we report the p*K*_a_ dependence of the acid reactivity with a series of POV-alkoxide clusters, [V_6_O_7_(OMe)_12_]^*n*^ (*n* = 2–, 1–, 0). The three clusters are identical in structure, differing from one another only in the oxidation state. Our findings demonstrate that despite possessing lower overall charges for the metal oxide cluster, terminal oxido moieties in POV-alkoxides exhibit sufficient basicity in an organic solvent across multiple charge states to bind and react with protons. Taken as a whole, this work asserts the role that the electronic structure (i.e., oxidation-state distribution) plays in dictating the cluster basicity, providing design criteria for the development of metal oxide nanomaterials with regioselective and tunable proton uptake characteristics.

## Results and Discussion

### p*K*_a_ Dependence of the Reactivity of **1-V**^**IV**^_**6**_**O**_**7**_^**2–**^ with Protons

**Scheme 2:**
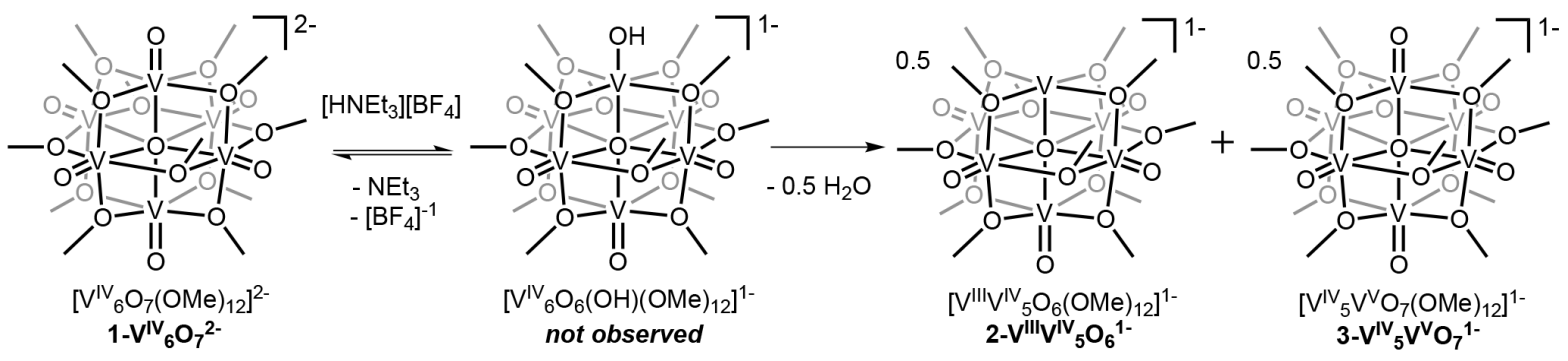
Acid-Driven Oxygen-Atom-Vacancy Formation via the Reaction of **1-V**^**IV**^_**6**_**O**_**7**_^**2–**^ with [HNEt_3_][BF_4_] [p*K*_a_(MeCN) = 18.82]

To deepen our understanding of the thermochemistry of proton uptake at the surface of POV-alkoxide clusters, we set out to elucidate the p*K*_a_ dependence of acid-induced oxygen-atom-vacancy formation. We opted to shift our focus from the POV-ethoxide cluster to its methoxide-substituted congener, **1-V**^**IV**^_**6**_**O**_**7**_^**2–**^, to minimize activation barriers associated with proton transfer as a result of steric clashes between organic acids and the surface of the cluster (note that previous work has established that the electronic structure of the hexavanadate core is conserved regardless of the identity of the bridging alkoxide ligand^41−44^). To confirm that complex **1-V**^**IV**^_**6**_**O**_**7**_^**2–**^ reacts similarly to [V_6_O_7_(OEt)_12_]^2–^ in the presence of 1 equiv of an organic acid, we first investigated the reactivity of the POV-methoxide cluster with [HNEt_3_][BF_4_] [p*K*_a_(MeCN) = 18.82].^45^ Instantaneous conversion to a mixture of the oxygen-deficient POV-methoxide cluster [V^III^V^IV^O_6_(OMe)_12_]^1−^ (**2-V**^**III**^**V**^**IV**^_**5**_**O**_**6**_^1^^**–**^) and the 1e^–^ fully oxygenated assembly [V^IV^_5_V^V^(OMe)_12_]^1−^ (**3-V**^**IV**^_**5**_**V**^**V**^**O**_**7**_^**1–**^) was observed (Scheme 2 and Figures S1 and S2). This outcome mirrors the reactivity reported previously for the POV-ethoxide cluster and thus confirms analogous behavior of the two distinct organofunctionalized assemblies in the presence of a weak organic acid.

**Figure 1:**
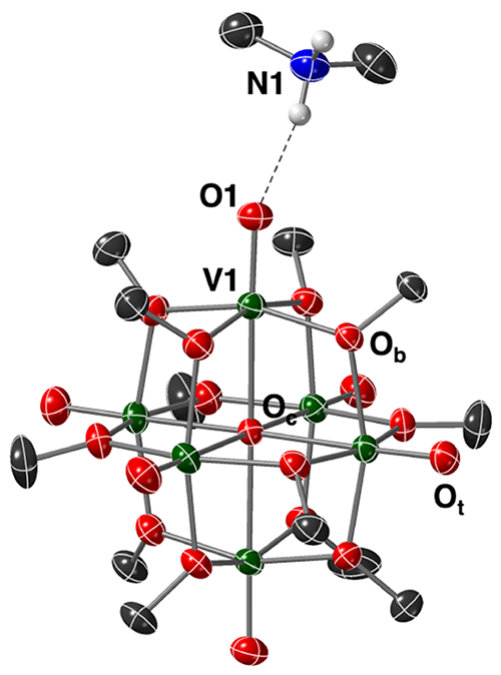
Molecular structure of [H_2_NMe_2_][^n^Bu_4_N][V_6_O_7_(OMe)_12_] shown with 30% probability ellipsoids. The tetrabutylammonium cation, cocrystallized solvent molecules, and selected hydrogen atoms have been removed for clarity. Key: dark-green ellipsoids, V; red ellipsoids, O; dark-gray ellipsoids, C; blue ellipsoids, N; white spheres, H.

As described in the introduction of this paper, the proposed mechanism for acid-induced defect formation in POV-alkoxide clusters invokes the formation of a transient hydroxide-substituted assembly, [V_6_O_6_(OH)(OMe)_12_]^1−^, via protonation of a surface vanadyl. Additional evidence supporting this mechanism was serendipitously obtained while screening the reactivity of **1-V**^**IV**^_**6**_**O**_**7**_^**2–**^ with organic acids of disparate strengths (*vide infra*). When **1-V**^**IV**^_**6**_**O**_**7**_^**2–**^ was reacted with 1 equiv of [H_2_NMe_2_][Cl] (p*K*_a_ = 19.03)^45^ in acetonitrile (MeCN), a teal blue precipitate formed. Crystals suitable for X-ray analysis were obtained by slow diffusion of diethyl ether (Et_2_O) into a concentrated methanol solution of the precipitate. Refinement of the crystallographic data revealed cation exchange, affording the dianionic POV-alkoxide cluster as a salt with a molecular formula of [H_2_NMe_2_][^n^Bu_4_N][V_6_O_7_(OMe)_12_] (Figure 1). Notably, the dimethylammonium cation is engaged in two types of hydrogen-bonding interactions, bridging two clusters to form a dimer. One proton of the organic acid forms a single hydrogen bond with a terminal oxido moiety, while the second proton is bifurcated between two adjacent bridging alkoxide ligands at the surface of another cluster molecule. The hydrogen-bonding motif is mirrored between the two clusters, with two organic acid molecules bridging two discrete hexavanadate anions.

The interactions with the bridging alkoxide ligands are notable but not totally unprecedented. While the proposed mechanism of cluster protonation is framed under the assumption that the alkoxide ligands will prevent cation pairing interactions at bridging oxygen atoms within the assembly, previous work from our laboratory investigating the interactions of POV-alkoxide clusters with alkali ions has shown that these oxygen atoms possess some degree of nucleophilic character.^46^ In the case of [H_2_NMe_2_][^n^Bu_4_N][V^IV^_6_O_7_(OMe)_12_], the O_b_···N1 distances of 2.874(3) and 2.967(3) Å are elongated in comparison to that between the dimethylammonium cation and the terminal oxido moiety [O1···N1 = 2.818(3) Å]. This observation suggests that the hydrogen-bonding interactions with the bridging alkoxide groups are weaker than those formed with terminal oxido groups. We hypothesize that these weak hydrogen-bonding interactions with the bridging alkoxide ligands are able to form as a result of the lack of steric bulk of the dimethylammonium cation, and are significant to the stability of the cluster–organic acid ion pair [as discussed below, the addition of *N*,*N*,*N*′,*N*′-tetramethylpropylenediammonium tetrafluoroborate ([HTMPDA][BF_4_]), an organic acid with a higher p*K*_a_ and increased steric bulk, results in conversion of the starting material]. Upon heating of a sample of [H_2_NMe_2_][^n^Bu_4_N][V^IV^_6_O_7_(OMe)_12_] in MeCN, partial conversion to a 1:1 mixture of **2-V**^**III**^**V**^**IV**^_**5**_**O**_**6**_^**1–**^ and **3-V**^**IV**^_**5**_**V**^**V**^**O**_**7**_^**1–**^ is observed (Figure S3). We hypothesize that the introduction of heat disrupts the hydrogen-bonding interactions with the face of the cluster, driving protonation of the cluster surface. As such, we infer that the hydrogen-bonding interactions between the ammonium salt and the oxygen atoms of bridging alkoxide ligands are not influential in the general reactivity of **1-V**^**IV**^_**6**_**O**_**7**_^**2-**^ with the other organic acids reported in this work.

The existence of a hydrogen bond between the dimethylammonium cation and a vanadyl moiety is a significant finding, as this result offers additional support for the proposed mechanism of acid-induced defect formation involving protonation of a terminal oxido ligand. The O1··· N1 interaction of 2.818(3) Å can be classified as a hydrogen bond of moderate strength, characteristic of a primarily electrostatic interaction between the organic acid and cluster surface (Table 1).^47^ Notably, the V1=O1 bond distance is slightly elongated [1.6227(17) Å vs Vn=O_t_ (avg; **1-V**^**IV**^_**6**_**O**_**7**_^**2–**^) = 1.606(1) Å] in the presence of an organic acid, albeit not to the extent consistent with formal reduction of the V=O_t_ bond.^35^

**Table 1:** Selected Bond Distances (Å) of [^n^Bu_4_N][V_6_O_7_(OMe)_12_]^33^ and [H_2_NMe_2_][^n^Bu_4_N][V_6_O_7_(OMe)_12_]

bond	**1-V**^**IV**^_**6**_**O**_**7**_^**2–**^	{[H_2_NMe_2_][V_6_O_7_(OMe)_12_]}^1−^
Vn–O_t_ (avg)	1.606(1) (*n* = 1–6)	1.6025(33) (*n* = 2–6)
V1–O1		1.6227(17)
O1··· H		1.97
O1··· N1		2.818(3)
O_b_···H		2.18, 2.20
O_b_···N1		2.874(3), 2.967(3)

Given the scarcity of proton interactions with terminal oxido ligands in POM research, we became interested in elucidating the p*K*_a_ dependence of proton interactions with terminal vanadyl moieties within POV-alkoxide clusters. Unfortunately, the instability of [V_6_O_6_(OH)(OMe)_12_]^1−^ prohibits direct measurement of the acidity of the OH bond.^48−51^ Instead, we invoke the quantification of disproportionation products as a means of assessing the extent of reaction for a given acid/cluster charge combination, under the assumption that the hydroxide-substituted assembly converts quantitatively to a 1:1 mixture of **2-V**^**III**^**V**^**IV**^_**5**_**O**_**6**_^**1–**^ and **3-V**^**IV**^_**5**_**V**^**V**^**O**_**7**_^**1–**^. In so doing, we can determine the strength of the organic acid required to react with half of the dianionic cluster by calculating the p*K*_a_ of an organic acid whose addition to **1-V**^**IV**^_**6**_**O**_**7**_^**2–**^ would yield a 2:1:1 speciation of **1-V**^**IV**^_**6**_**O**_**7**_^**2–**^, **2-V**^**III**^**V**^**IV**^_**5**_**O**_**6**_^**2–**^, and **3-V**^**IV**^_**5**_**V**^**V**^**O**_**7**_^**2–**^, respectively. For the purpose of this work, we describe this value as the p*K*_a_ of the conjugate acid of **1-V**^**IV**^_**6**_**O**_**7**_^**2–**^, the transient hydroxide-functionalized assembly, [V_6_O_6_(OH)(OMe)_12_]^1−^, and use this value to describe the basicity of the cluster surface.

To quantify the strength of the acid required for surface activation of **1-V**^**IV**^_**6**_**O**_**7**_^**2–**^, we explored the reactivity of the reduced assembly with a series of organic acids, all of which possess acid dissociation constants that have been reported in MeCN (Table S1).^45,52−55^ As discussed above, the ^1^H NMR spectrum of the crude reaction mixture of **1-V**^**IV**^_**6**_**O**_**7**_^**2–**^ with [HNEt_3_][BF_4_] reveals four paramagnetically shifted and broadened resonances (Figure S1); three of these signals correspond to the oxygen-deficient assembly **2-V**^**III**^**V**^**IV**^_**5**_**O**_**6**_^1^^**–**^ (δ = 25.3, 23.9, and −15.6 ppm), while the fourth signal corresponds to the fully oxygenated species **3-V**^**IV**^_**5**_**V**^**V**^**O**_**7**_^**1–**^ (δ = 23.4 ppm). It is important to note that the signal assigned to the protons of the methoxide substituents of the starting material, **1-V**^**IV**^_**6**_**O**_**7**_^**2–**^, is observed at a similar chemical shift (δ = 23.9 ppm). The overlap of the paramagnetically broadened and shifted resonances corresponding to the fully oxygenated species in the mono- and dianionic charge states convolutes analysis of the resultant ^1^H NMR spectrum in the case of a reaction mixture that has not reached completion (Figures S4 and S5). This necessitates the use of an alternative approach to quantify product speciation and, accordingly, the extent of reaction between a given acid and **1-V**^**IV**^_**6**_**O**_**7**_^**2–**^.

Fortuitously, the electronic absorption spectra of the three species in this reaction are distinct, allowing for determination of the quantity of relevant compounds in solution following the addition of acid (Figure S6). The fully oxygenated, mixed-valent, monoanionic assembly,**3-V**^**IV**^_**5**_**V**^**V**^**O**_**7**_^**1–**^ features diagnostic absorptions centered at 386 nm (ε = 3694 M^–1^ cm^–1^) and 1000 nm (ε = 525 M^–1^ cm^–1^). In contrast, complexes **1-V**^**IV**^_**6**_**O**_**7**_^**2–**^ and **2-V**^**III**^**V**^**IV**^_**5**_**O**_**6**_^1**–**^ exhibit comparatively weaker molar absorptivities at these wavelengths (**1-V**^**IV**^_**6**_**O**_**7**_^**2-**^, ε_386_ = 248 M^–1^ cm^–1^ and ε_1000_ = 120 M^–1^ cm^–1^; **2-V**^**III**^**V**^**IV**^_**5**_**O**_**6**_^1**–**^, ε_386_ = 898 M^–1^ cm^–1^ and ε_1000_ = 58 M^–1^ cm^–1^). As such, monitoring the change in the absorptivity at either of these wavelengths following the addition of 1 equiv of acid can serve as a spectroscopic handle to determine the extent of reaction.

**Figure 2:**
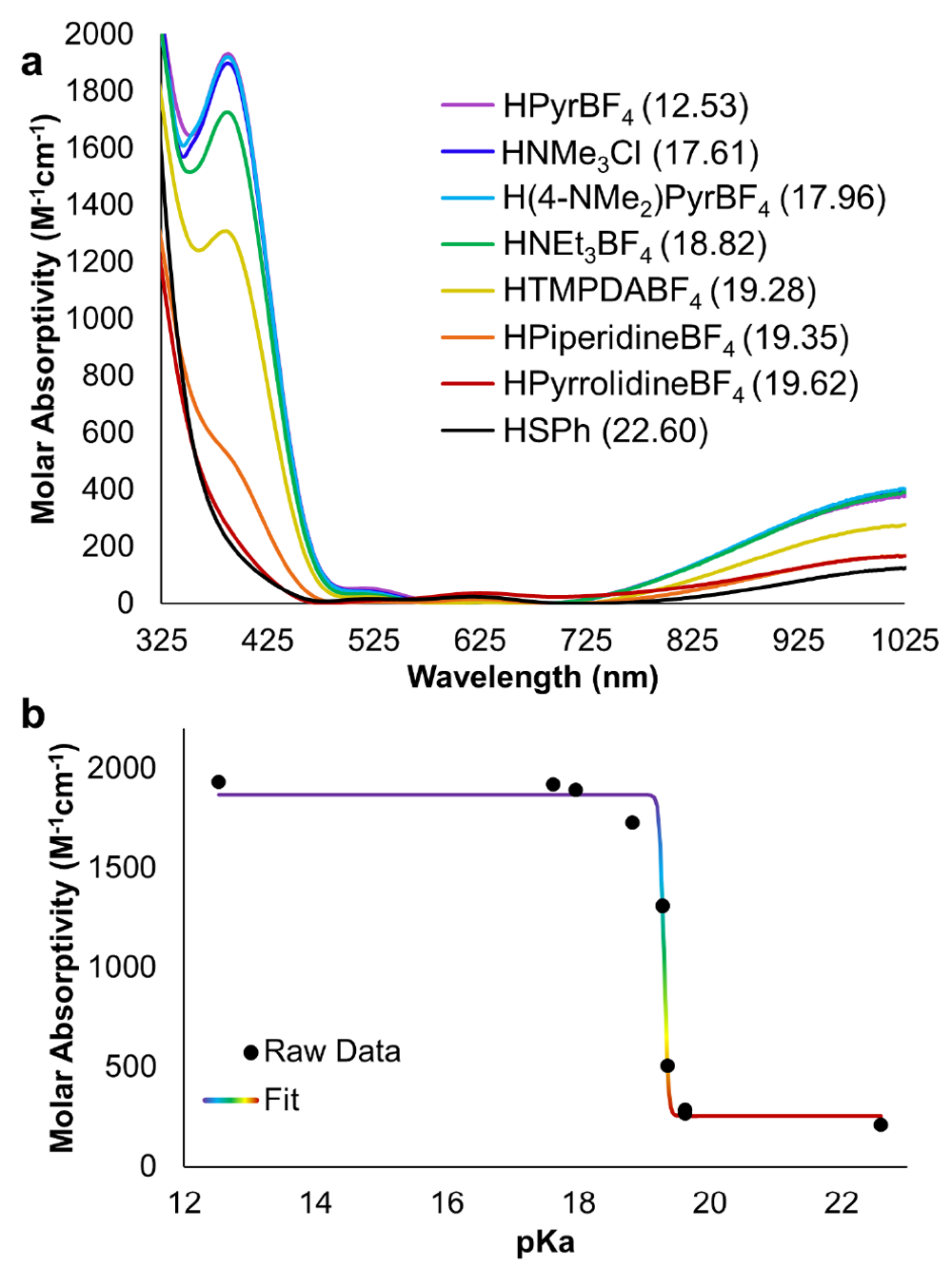
(a) Electronic absorption spectra collected for reactions between **1-V**^**IV**^_**6**_**O**_**7**_^**2–**^ and 1 equiv of organic acids in MeCN at 21 °C. (b) Plot of the molar absorptivity of the reaction solution at 386 nm as a consequence of the strength of the added organic acid.

To probe the acid dependence on defect formation, we evaluated the absorption spectrum of the crude reaction mixture upon the addition of 1 equiv of a series of organic acids (p*K*_a_ = 12.53–22.60) to complex **1-V**^**IV**^_**6**_**O**_**7**_^**2–**^ (Figure 2 and Table S2). The molar absorptivities at 386 and 1000 nm are unchanged following the addition of the weakest acids evaluated (p*K*_a_ values ranging from 19.62 to 22.60). The addition of marginally stronger acids (p*K*_a_ = 18.82–19.35) yields a large increase in the absorbance at both wavelengths, with stronger acids (p*K*_a_ = 12.53–17.96) displaying a minimal effect on further reactivity. The plateau in the molar absorptivity indicates complete consumption of acidic protons, with p*K*_a_ values of less than 17.96. The generated plot of the molar absorptivity at 386 nm versus acid strength yields a sigmoidal relationship, which can be fit with the function
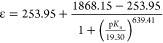
1where 253.95 and 1868.15 M^–1^ cm^–1^ are the lower and upper bounds of the function, respectively, p*K*_a_ is the strength of the applied acid, and 19.30 and 639.41 are constants derived from the fit of the curve (eq 1 and Figure 2b). Using Beer’s law, the molar absorptivity at 386 nm of the 2:1:1 speciation produced when a proton source activates half of the available **1-V**^**IV**^_**6**_**O**_**7**_^**2–**^, resulting in a reaction mixture containing a 2:1:1 ratio of **1-V**^**IV**^_**6**_**O**_**7**_^**2–**^, **2-V**^**III**^**V**^**IV**^_**5**_**O**_**6**_^1**–**^, and **3-V**^**IV**^_**5**_**V**^**V**^**O**_**7**_^**1–**^, was determined to be 1272 M^–1^ cm^–1^ (Table S2; see the Supporting Information for additional details pertaining to this calculation). Solving the p*K*_a_ value of the organic acid, following whose addition results in conversion of half of the dianionic assembly, is achieved using this molar absorptivity (eq 1 and Figure 2b). On the basis of the assumptions described above, we correlate this value to the p*K*_a_ value of the transient hydroxide-functionalized assembly [V_6_O_6_(OH)(OMe)_12_]^1−^ in MeCN (p*K*_a_ = 19.28). A plot was generated using the molar absorptivities for the aforementioned reactions at 1000 nm to confirm the product speciation; using this wavelength, a p*K*_a_ value of [V_6_O_6_(OH)(OMe)_12_]^1−^ of 19.31 was found, within error of that resolved using the absorption data at 386 nm (Figure S7 and Table S2).

The basicity of this cluster is remarkable compared to the proton affinity established for other POMs in MeCN. Previous reports on the protonation of POMs have revealed that clusters bearing large negative charges (e.g., [S_2_W_18_O_62_]^4–^, [a-PW_12_O_40_]^3–^, and [β-PW_12_O_40_]^3–^)^18,24,25,56^ have very low affinity for protons. This observation is counterintuitive, as one would expect that the increase in electrostatic forces would play a substantial role in proton uptake. We believe the high proton affinity of our modestly charged cluster can be explained by two features of the assembly. First, the POV-alkoxide is comprised of group V metal ions, which are known to be more basic than their group VI counterparts.^27,57−59^ This has been reflected in several reports on the protonation of vanadium- and niobium-functionalized polyoxotungstates and molybdates.^19−21,27,58−62^ In all cases, protonation of the heterometallic site(s) is preferred, as revealed by structural, electrochemical, and theoretical analyses. This is due to the lower oxidation state of the *d*^0^ group V ion, which is unable to compensate for the charge of the bound O^2–^ ligands, as demonstrated on isostructural, homometallic POMs comprised of different metal ions.^57^ The increased partial negative charge resides on the oxide ligands surrounding the group V ion(s) in the heterometallic structure, resulting in increased basicity at these sites.

In addition, the high proportion of vanadium(IV) centers within the POV-alkoxide promotes enhanced proton uptake in this assembly in comparison to other POMs. POMs are typically isolated with fully oxidized metal ions. The *d*^0^ metal centers withdraw electron density from coordinated oxygen atoms, reducing the nucleophilicity of the surface oxido moieties despite the large negative charge of the assembly. In contrast, the dianionic form of the cluster is composed entirely of tetravalent (*d*^1^) vanadium ions. Because all of the vanadium ions comprising the cluster core contain one d electron, the metal centers are more electropositive than their oxidized congener.^63,64^ This further increases the electron density at the terminal oxido moieties, rendering these sites more reactive with protons from the surrounding medium.

### Charge-State Dependence of the Surface Basicity of POV-alkoxide Clusters

The methoxide-substituted POV-alkoxide cluster has been structurally and spectroscopically characterized in four distinct charge states, namely, complexes **1-V**^**IV**^_**6**_**O**_**7**_^**2–**^, **3-V**^**IV**^_**5**_**V**^**V**^**O**_**7**_^**1–**^, [V^IV^_4_V^V^_2_O_7_(OMe)_12_]^0^ (**4-V**^**IV**^_**4**_**V**^**V**^_**2**_**O**_**7**_^**0**^), and [V^IV^_3_V^V^_3_O_7_(OMe)_12_]^1+^ (**5-V**^**IV**^_**3**_**V**^**V**^_**3**_**O**_**7**_^**1+**^).^33,41,43^ Interested in interrogating the charge-state dependence on proton uptake, we next investigated the reactivity of organic acids with a series of POV-alkoxides in higher oxidation states. These studies are predicated on the assumption that higher oxidation states of the POV-alkoxide cluster (e.g., [V_6_O_7_(OMe)_12_]^*n*^; *n* = 1–, 0) proceed via a similar mechanism of surface protonation of a vanadyl moiety to generate [V_6_O_6_(OH)(OMe)_12_]^*n*+1^, followed by disproportionation to generate [V_6_O_7_(OMe)_12_]^*n*+1^ and [V_6_O_6_(OMe)_12_]^*n*+1^. We opted to narrow the scope of our investigations to the reactivity of complexes **3-V**^**IV**^_**5**_**V**^**V**^**O**_**7**_^**1–**^ and **4-V**^**IV**^_**3**_**V**^**V**^_**2**_**O**_**7**_^**0**^ with organic acids because the proposed products of proton uptake have been shown to be stable in MeCN.^33,41,43,65,66^

**Figure 3:**
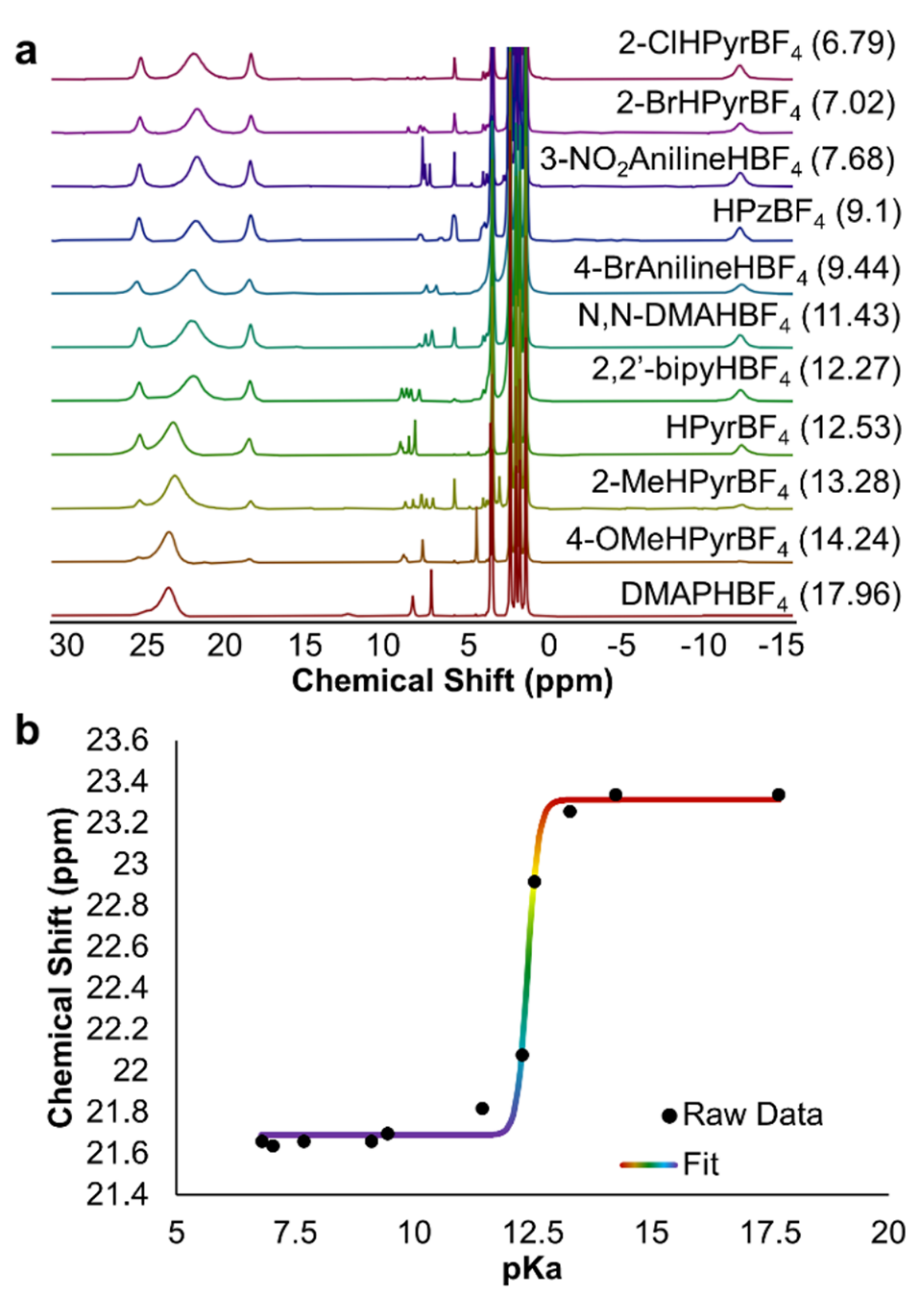
(a) ^1^H NMR spectra collected for reactions between **3-V**^**IV**^_**5**_**V**^**V**^**O**_**7**_^**1–**^ and 1 equiv of organic acids in MeCN-*d*_3_ at 21 °C. (b) Plot of the chemical shift of the fully oxygenated Lindqvist cluster in solution as a consequence of the strength of the added organic acid.

Upon exposure of the monoanionic POV-alkoxide complex **3-V**^**IV**^_**5**_**V**^**V**^**O**_**7**_^**1–**^ to 1 equiv of [HNEt_3_][BF_4_] (p*K*_a_ = 18.82), the anticipated conversion to a 1:1 mixture of the 1e^–^ species **4-V**^**IV**^_**4**_**V**^**V**^_**2**_**O**_**7**_^**0**^ and the oxygen-atom-deficient assembly [V_6_O_6_(OMe)_12_]^0^ (**6-V**^**III**^**V**^**IV**^_**4**_**V**^**V**^**O**_**6**_^**0**^) was not observed. Instead, signals assigned to the starting materials, **3-V**^**IV**^_**5**_**V**^**V**^**O**_**7**_^**1–**^ and [HNEt_3_][BF_4_], were noted, suggesting no interaction between the cluster and this proton source (Figure S8). Hypothesizing that the change in cluster charge state may be associated with a decreased basicity of the surface vanadyl moieties, we opted to investigate the reactivity of **3-V**^**IV**^_**5**_**V**^**V**^**O**_**7**_^**1–**^ with a stronger acid. When 1 equiv of pyrazolium tetrafluoroborate ([HPz][BF_4_]; p*K*_a_ = 9.1)^45^ was added to the monoanionic POV-alkoxide cluster, the resultant ^1^H NMR spectrum contains chemical shifts corresponding to the 1e^–^ assembly **4-V**^**IV**^_**4**_**V**^**V**^_**2**_**O**_**7**_^**0**^ (δ = 21.7 ppm) and the neutral, oxygen-deficient cluster **6-V**^**III**^**V**^**IV**^_**4**_**V**^**V**^**O**_**6**_^**0**^ (δ = 25.3, 18.2, and −12.6 ppm; Figure 3).

To quantify the basicity of the cluster surface, we explored the reactivity of **3-V**^**IV**^_**5**_**V**^**V**^**O**_**7**_^1^^**–**^ with a series of organic acids of varying strength (p*K*_a_ = 6.79–17.96). In the case of the more oxidized assembly, an alternative analytical approach was needed, as all three clusters contained within the reaction mixture (complexes **3-V**^**IV**^_**5**_**V**^**V**^**O**_**7**_^**1–**^, **4-V**^**IV**^_**4**_**V**^**V**^**O**_**7**_^**0**^, and **6-V**^**III**^**V**^**IV**^_**4**_**V**^**V**^**O**_**6**_^**0**^) possess mixed-valent [vanadium(IV)/vanadium(V)] electronic structures that manifest in intense absorptions at ∼390 and ∼1000 nm.^65−67^ Fortunately, products formed upon acid-induced oxygen-atom-vacancy formation from complex **3-V**^**IV**^_**5**_**V**^**V**^**O**_**7**_^**–**^ exhibit distinct chemical shifts(Figure S9); we thus hypothesized that all of the relevant species in solution following acid addition could be quantified via ^1^H NMR spectroscopy.

To our surprise, in monitoring of the ^1^H NMR spectra of a series of reactions exposing complex **3-V**^**IV**^_**5**_**V**^**V**^**O**_**7**_^**1–**^ to 1 equiv of various organic acids, only four paramagnetically broadened and shifted signals were observed as opposed to the expected five signals (Figure 3a). Three of these signals correspond to the expected oxygen-deficient assembly, complex **6-V**^**III**^**V**^**IV**^_**4**_**V**^**V**^**O**_**6**_^**0**^, and appear to increase in intensity proportionately with the strength of the added organic acid. Interestingly, the fourth resonance is observed between 21.7 and 23.4 ppm, corresponding to the reported chemical shift of neither **3-V**^**IV**^_**5**_**V**^**V**^**O**_**7**_^**1–**^ nor **4-V**^**IV**^_**4**_**V**^**V**^_**2**_**O**_**7**_^**0**^. As the p*K*_a_ value of the added organic acid decreases, the chemical shift of the broad resonance moves upfield, toward the established value of **4-V**^**IV**^_**4**_**V**^**V**^_**2**_**O**_**7**_^**0**^ (δ = 21.7 ppm), settling when the p*K*_a_ value of the organic acid added is less than or equal to 9.44 (Figure 3a). In addition, upon assessment of the relative integration of the paramagnetically shifted resonance at 18.2 ppm compared with those at 25.3 and 23.4–21.7 ppm (taken together because of overlap in reactions with weak acids), it is observed that the speciation in acid addition reactions differs very little in reactions between **3-V**^**IV**^_**5**_**V**^**V**^**O**_**7**_^**1–**^ and acids with p*K*_a_ values below 9.44 (Table S3). The relative integrations of these NMR signals for reactions with strong acids (p*K*_a_ < 9.44) presents an approximate 1:4 ratio between the peak at 18.2 and the two more-downfield shifts, respectively, matching what is expected for a 1:1 mixture of **4-V**^**IV**^_**4**_**V**^**V**^_**2**_**O**_**7**_^**0**^ and **6-V**^**III**^**V**^**IV**^_**4**_**V**^**V**^**O**_**6**_^**0**^. The lack of significant differences in the relative integrations for spectra of reactions with strong acids indicates that the addition of an organic acid with a p*K*_a_ value lower than 9.44 would be expected to result in complete conversion of complex **3-V**^**IV**^_**5**_**V**^**V**^**O**_**7**_^1^^**–**^ and that the region where the p*K*_a_ value of the acid dictates the extent of conversion should fall between 9.44 and 14.24. Whilea comparison of the relative integrations of paramagnetic protons provides general information into this p*K*_a_-dependent reactivity window, we do note that because of the likely differences in the relaxation time for each resonance comparing signal integrations is not an accurate measure of the extent of reactivity of the parent POV-alkoxide.

**Figure 4:**
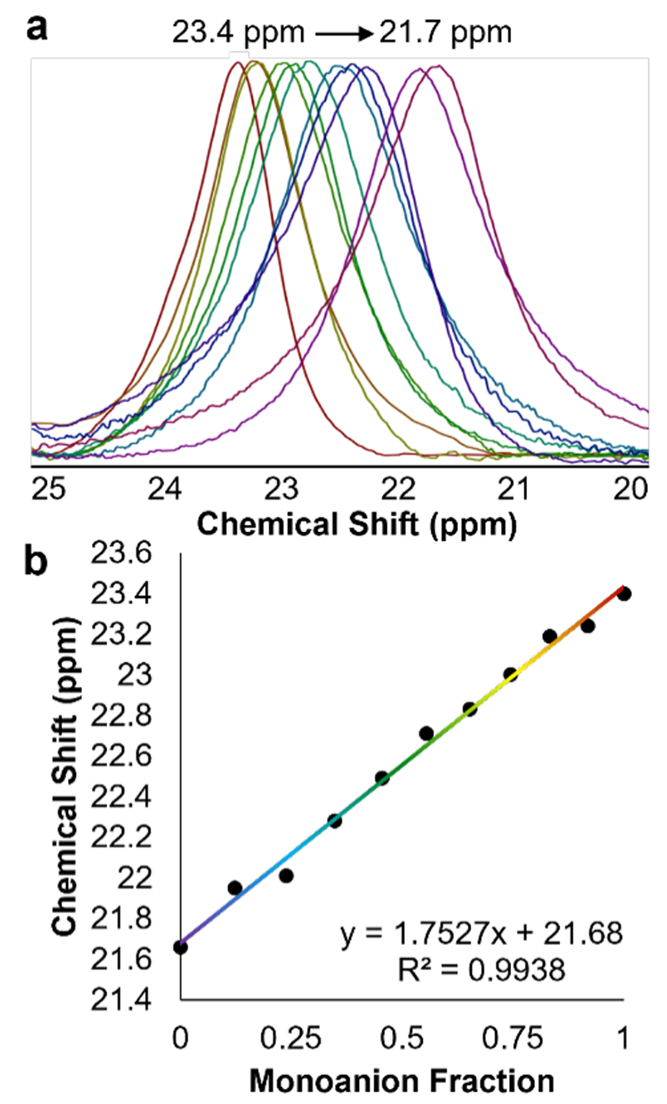
(a) ^1^H NMR spectra collected for charge-state mixtures between **3-V**^**IV**^_**5**_**V**^**V**^**O**_**7**_^**1–**^ and **4-V**^**IV**^_**4**_**V**^**V**^_**2**_**O**_**7**_^**0**^ in MeCN-*d*_3_ at 21 °C. (b) Plot of the chemical shift of the fully oxygenated Lindqvist cluster in solution as a consequence of the ratio of **3-V**^**IV**^_**5**_**V**^**V**^**O**_**7**_^**1–**^ present in a given sample.

The observed fluxionality of the chemical shift of the single broad resonance, however, presents an opportunity to quantify the contents of the reaction mixture. We hypothesized that electron transfer exceeding the time scale of the ^1^H NMR experiment between the unreacted monoanionic POV-alkoxide cluster **3-V**^**IV**^_**5**_**V**^**V**^**O**_**7**_^**1–**^ and the 1e^–^ assembly formed following cluster protonation, **4-V**^**IV**^_**4**_**V**^**V**^_**2**_**O**_**7**_^**0**^, may explain the observed behavior.^42−44,68−70^ Thus, we ran a series of control experiments targeting an understanding of the consequences of having a mixture of charge states of the POV-alkoxide cluster in MeCN-*d*_3_ on the resultant ^1^H NMR spectrum. Various ratios of **3-V**^**IV**^_**5**_**V**^**V**^**O**_**7**_^**1–**^ and **4-V**^**IV**^_**4**_**V**^**V**^_**2**_**O**_**7**_^**0**^ were prepared, and the resultant ^1^H NMR spectra were analyzed (Figure 4; see the Supporting Information for additional details). A comparison of the spectral data reveals, in all cases, a single broad resonance, as opposed to two distinct signals corresponding to **3-V**^**IV**^_**5**_**V**^**V**^**O**_**7**_^**1–**^ and **4-V**^**IV**^_**4**_**V**^**V**^_**2**_**O**_**7**_^**0**^. This finding suggests that rapid electron transfer occurs between the two disparate charge states of the cluster at room temperature. Notably, the signal shifts upfield as the concentration of **4-V**^**IV**^_**4**_**V**^**V**^_**2**_**O**_**7**_^**0**^ increases relative to that of **3-V**^**IV**^_**5**_**V**^**V**^**O**_**7**_^**1–**^. A plot of the chemical shift as a function of the ratio of **3-V**^**IV**^_**5**_**V**^**V**^**O**_**7**_^**1–**^ to **4-V**^**IV**^_**4**_**V**^**V**^_**2**_**O**_**7**_^**0**^ reveals a linear correlation (Figure 4b), allowing for quantification of the relative proportions of the neutral and monoanionic POV-alkoxide clusters in solution from the chemical shift of the resonance. Similar phenomena involving the comproportionation of disparate oxidation states of POMs on the NMR time scale have been observed in several reports; heteronuclear (e.g., ^31^P^71,72^ and ^27^Al^73^) NMR as well as electron paramagnetic resonance spectroscopies on charge-state mixtures of polyoxotungstates have revealed intermolecular electron exchange.^74^

With this information in hand, we are able to interpret the data collected in the aforementioned experiments targeting resolution of the surface basicity of **3-V**^**IV**^_**5**_**V**^**V**^**O**_**7**_^**1–**^. Using the calibration curve established by correlating the chemical shift for clusters of mixed charge states to the ratio of **3-V**^**IV**^_**5**_**V**^**V**^**O**_**7**_^**1–**^ to **4-V**^**IV**^_**4**_**V**^**V**^_**2**_**O**_**7**_^**0**^ in solution, we can determine the relative concentrations of species in the resultant reaction mixture. To determine the p*K*_a_ value of the protonated form of complex **3-V**^**IV**^_**5**_**V**^**V**^**O**_**7**_^**1–**^, a neutral hydroxide-substituted vanadium oxide assembly, [V_6_O_6_(OH)(OMe)_12_]^0^, a plot of the chemical shift of the signal corresponding to [V_6_O_7_(OMe)_12_]^*n*^ (*n* = 1–, 0) versus p*K*_a_ of the added organic acid was generated (Figure 3b). This plot reveals a sigmoidal relationship, which can be fit by the following equation:
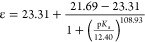
2where 23.31 and 21.69 ppm are the upper and lower bounds of the function, respectively, p*K*_a_ is the strength of the applied acid, and 12.40 and 108.93 are constants. The p*K*_a_ value for cluster protonation of 12.51 is determined using the chemical shift for a 2:1 mixture of **3-V**^**IV**^_**5**_**V**^**V**^**O**_**7**_^**–**^ and **4-V**^**IV**^_**4**_**V**^**V**^_**2**_**O**_**7**_^**0**^ (22.86 ppm).

**Figure 5:**
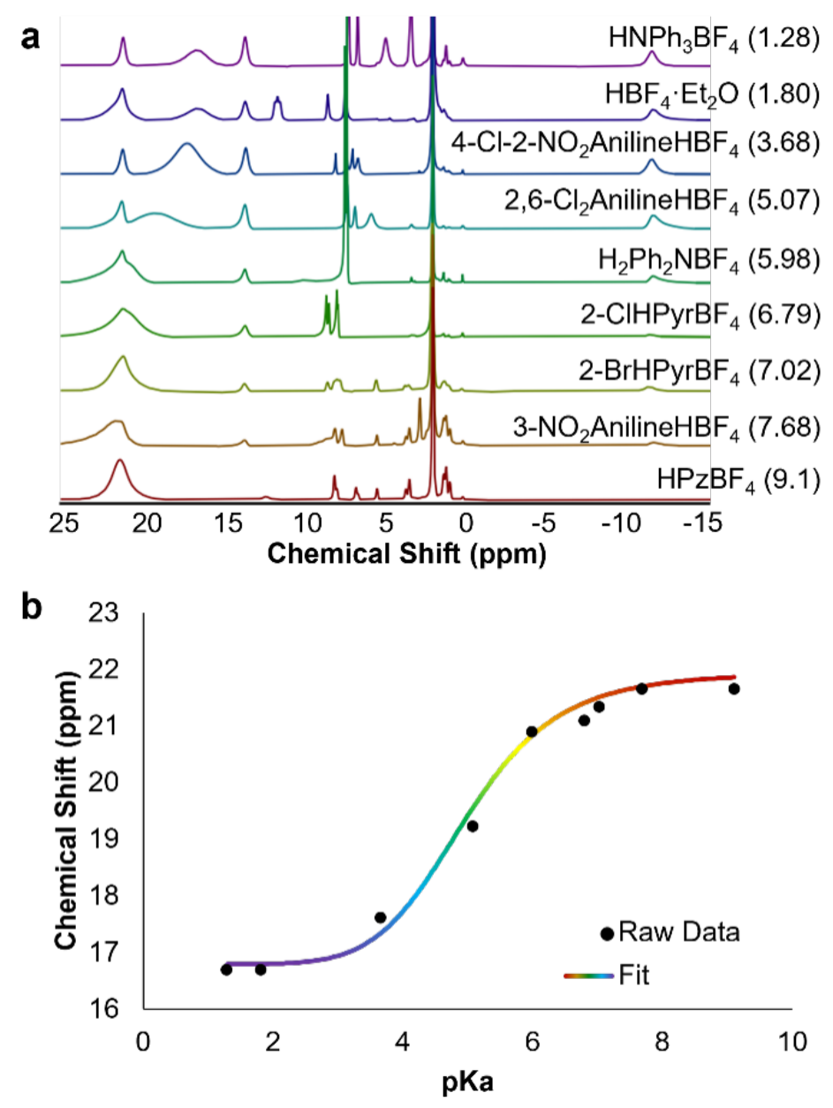
(a) ^1^H NMR spectra collected for reactions between **4-V**^**IV**^_**4**_**V**^**V**^_**2**_**O**_**7**_^**0**^ and 1 equiv of organic acids in MeCN-*d*_3_ at 21 °C. (b) Plot of the chemical shift of the Lindqvist cluster in solution as a consequence of the strength of the added organic acid.

Next, we investigated the reactivity of complex **4-V**^**IV**^_**4**_**V**^**V**^_**2**_**O**_**7**_^**0**^ with organic acids. We hypothesized that the increase in the charge state to the neutral assembly would similarly necessitate the addition of stronger organic acids to drive the protonation to completion. Indeed, complex **4-V**^**IV**^_**4**_**V**^**V**^_**2**_**O**_**7**_^**0**^ does not react with [HNEt_3_][BF_4_] (p*K*_a_ = 18.82) or [HPz][BF_4_] (p*K*_a_ = 9.1). However, when complex **4-V**^**IV**^_**4**_**V**^**V**^_**2**_**O**_**7**_^**0**^ is exposed to triphenylammonium tetrafluoroborate ([HNPh_3_][BF_4_]; p*K*_a_ = 1.28),^45^ we observe the formation of a 1:1 mixture of [V_6_O_7_(OMe)_12_]^1^^+^ (**5-V**^**IV**^_**3**_**V**^**V**^_**3**_**O**_**7**_^**1+**^; δ = 16.7 ppm) and three additional signals at 21.4, 13.7, and −11.8 ppm (Figure 5). Given the stoichiometry of the reaction, we hypothesized that this unfamiliar set of resonances in the ^1^H NMR spectrum of the crude reaction mixture might correspond to the cationic, oxygen-atom-deficient POV-alkoxide cluster [V_6_O_6_(OMe)_12_(MeCN)][BF_4_] (**7-V**^**III**^**V**^**IV**^_**3**_**V**^**V**^_**2**_**O**_**6**_^**1+**^). Indeed, an independent synthesis of this complex was achieved by the oxidation of **6-V**^**III**^**V**^**IV**^_**4**_**V**^**V**^**O**_**6**_^**0**^ with 1 equiv of AgBF_4_ in dichloromethane (DCM). The resultant ^1^H NMR spectrum reveals the same three-peak pattern as that observed in the acid addition reaction, albeit with a significant impurity peak at 21.7 ppm corresponding to **4-V**^**IV**^_**4**_**V**^**V**^_**2**_**O**_**7**_^**0**^, likely formed as a consequence of the reactive nature of oxygen-atom-deficient clusters with adventitious water (Figure S10).

With product speciation in hand, we were able to determine the acid strength required to react with 50% of the neutral cluster in solution (Figure 5). It is important to note that, again, rapid electron transfer between the neutral and cationic states of the fully oxygenated Lindqvist POV-alkoxide results in a shift of the ^1^H NMR signal for this complex (Figure S11 and see the Supporting Information for addition details). Analysis of the resultant ^1^H NMR spectra of a series of cluster/acid combinations produces a relationship similar to those in the previous two examples, where certain acids (p*K*_a_ = 7.02–9.1) do not produce any oxygen-atom defects and stronger proton donors (p*K*_a_ = 1.28–5.98) gradually convert **4-V**^**IV**^_**4**_**V**^**V**^_**2**_**O**_**7**_^**0**^ to a mixture of **5-V**^**IV**^_**3**_**V**^**V**^_**3**_**O**_**7**_^**1+**^ and the oxygen-deficient assembly **7-V**^**III**^**V**^**IV**^_**3**_**V**^**V**^_**2**_**O**_**6**_^**1+**^ (Figure 5a). Plotting the chemical shift of the signal corresponding to a mixture of [V_6_O_7_(OMe)_12_]^*n*^ (*n* = 0, 1+) versus p*K*_a_ of the added organic acid reveals the anticipated sigmoidal relationship, which is fit by the function
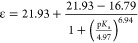
3where 21.93 and 16.79 ppm are the upper and lower bounds, respectively, p*K*_a_ is the applied acid strength, and 4.97 and 6.94 are constants (Figure 5b). The chemical shift of the expected 2:1 ratio of **4-V**^**IV**^_**4**_**V**^**V**^_**2**_**O**_**7**_^**0**^ and **5-V**^**IV**^_**3**_**V**^**V**^_**3**_**O**_**7**_^**1+**^ present after conversion of half the initial neutral cluster with an acid is 20.24 ppm; this value corresponds to a p*K*_a_ value for the purported protonated POV-alkoxide cluster of 5.51.

As described at the outset of this work, the experimentally determined p*K*_a_ values for the purported hydroxide functionalized POV-alkoxide clusters, [V_6_O_6_(OH)(OMe)_12_]^*n*^ (*n* = 1–, 0, 1+), can be used to describe the basicity of the parent, fully oxygenated assembly. Analysis of the data reported reveals a trend, with reduced derivatives of the organofunctionalized vanadium oxide assembly possessing a significantly increased affinity for protons in MeCN compared to their oxidized congeners. This result is consistent with previous studies describing the relative basicity of POMs across multiple charge states, in which cluster reduction has been shown to enhance proton association constants. For instance, Wedd and co-workers showed that reduction of the Wells–Dawson complex [S_2_W_18_O_62_]^4–^ in the presence of acidic protons yields anodically shifted redox waves, indicating PCET to the cluster surface. The reduced species [S_2_W_18_O_61_(OH)]^*n*^ (*n* = 5–, 6–, 7−) feature proton association constants of 2 × 10^–1^, 2 × 10^8^, and 3 × 10^13^ M^–1^, respectively, in MeCN.^18^ The large equilibrium constants for the most reduced variants of the cluster suggest that as electrons are added to the assembly, the surface of the cluster dramatically increases in its affinity for protons. Similar results have been noted for the α- and β-isomers of the Keggin-type polyoxotungstate [PW_12_O_40_]^3–^, where the addition of electron density to the assembly corresponds to an increased basicity of the cluster surface.^24,25,56^

As the POV-alkoxide cluster is oxidized, the number of vanadium(V) centers contained within the hexavanadate core increases, decreasing the average electropositivity of the V=O sites within the cluster. Therefore, because **3-V**^**IV**^_**5**_**V**^**V**^**O**_**7**_^1^^**–**^ and **4-V**^**IV**^_**4**_**V**^**V**^_**2**_**O**_**7**_^**0**^ contain one and two d^0^ metal centers, respectively, their average electropositivities will be lower than that of the fully reduced **1-V**^**IV**^_**6**_**O**_**7**_^**2–**^, resulting in observed decreases in the basicity for each electron removed. However, because both oxidized clusters are composed of a majority of reduced vanadium centers, the average electropositivity of the core metal ions facilitates measurable proton uptake to the modestly charge assemblies. This further supports the notion that the electronic structure [i.e., the ratio of vanadium(V)/vanadium(IV) ions contained within the vanadium oxide assembly] has a more substantial impact on the basicity of the polyoxovanadate surface (as opposed to molecular charge).

It is worth mentioning that in our previous work investigating charge-compensation reactions between POV-alkoxide clusters and alkali ions, we observed that these cations do not interact with the surface of the cluster in its monoanionic and neutral charge states.^46^ This observation renders the reactivity of the monoanionic and neutral POV-alkoxide clusters with protons particularly intriguing. We propose two possible, nonexclusive, explanations for the observed reactivity. The first pertains to the irreversibility of the protonation reaction. Generally, POM protonation has been shown to be a reversible equilibrium process able to be quantified by association constants extracted from electrochemical analyses.^18,24,25,56^ In our system, the acid–base equilibrium is disrupted by rapid disproportionation of the protonated cluster. Considering Le Chatelier’s principle, this drives the equilibrium to favor its protonated form. The irreversible proton transfer enhances the basicity of the more oxidized clusters, resulting in proton uptake for clusters that do not feature surface coordination of alkali ions. Separately, the reducibility of protons may facilitate proton uptake to modestly charged clusters. Alkali ions require high potentials to be reduced by an electron donor; for example, the potential of the Li^0/+^ couple is −3.05 V versus standard hydrogen electrode.^75^ Because the reduction of protons is accessible at much lower potentials and dependent on the strength of the proton donor,^76^ the POV-alkoxide cluster may more readily transfer electron density to the proton, resulting in the reactivity being better described as that of the vanadium oxide assembly with hydrogen atoms, diminishing the importance of electrostatic forces.

## Conclusions

In this work, we have quantified the basicity of a POV-alkoxide cluster in a variety of charge states in MeCN. This property is presented as the p*K*_a_ value of the transient hydroxide-terminated species [V_6_O_6_(OH)(OCH_3_)_12_]^*n*^ (*n* = 1–, 0, 1+), providing a thermochemical descriptor for acid-induced oxygen-atom-vacancy formation on the Lindqvist core. The molecular vanadium oxide studied in this work exhibits regioselectivity toward protonation at terminal oxido moieties, imparted by the saturation of bridging oxides with organic groups, giving rise to the formation of an atypical terminal vanadium hydroxide that rapidly converts to an oxygen-atom vacancy poised for downstream reactivity. Our findings indicate that the basicity of terminal oxido moieties at the surface of the assembly is primarily a consequence of vanadium-ion electropositivity and can be tailored by tuning the ratio of oxidized and reduced metal ions (∼7 p*K*_a_ units per electron lost). We also found that cluster interactions with protons vastly differ from those with alkali ions because of the chemical irreversibility of the protonation reaction as well as the redox activity of the added cationic species. Overall, the cluster’s structural and electronic characteristics serve as atomically precise descriptors for nanocrystalline metal oxide design criteria (i.e., capping ligand density and oxidation state distribution), suggesting that control over these features should allow for careful tuning of the material activity with protons.

## Experimental Section

### General Considerations

All manipulations were carried out in the absence of water and oxygen using standard Schlenk techniques or in a UniLab MBraun inert-atmosphere drybox under a N_2_ atmosphere. All glassware was oven-dried for a minimum of 4 h and cooled in an evacuated antechamber prior to use in the drybox. Solvents were dried and deoxygenated on a Glass Contour System (Pure Process Technology, LLC) and stored over activated 3 Å molecular sieves purchased from Fisher Scientific prior to use. [^n^Bu_4_N]BH_4_, AgOTf, AgBF_4_, a tetrafluoroboric acid ether complex, thiophenol, triphenylamine, 4-chloro-2-nitroaniline, 2,6-dichloroaniline, 3-nitroaniline, diphenylamine, pyrazole, 4-bromoaniline, 2,2′-bipyridine, trimethylammonium chloride, and dimethylammonium chloride were purchased from Sigma-Aldrich and used as received. 2-Chloropyridine, 2-bromopyridine, 4-*N*,*N*-dimethylaniline, pyridine, 2-methylpyridine, 4-methoxypyridine, 4-*N*,*N*-dimethylpyridine, triethylamine, *N*,*N*,*N*′,*N*′-tetramethyl-1,3-propylenediamine, piperidine, and pyrrolidine were purchased from Sigma-Aldrich, dried over KOH under an atmosphere of Ar, distilled, and stored over activated 3 Å molecular sieves prior to use. 4-Bromoaniline was purchased from Alpha Aesar and used as received. Complexes **3-V**^**IV**^_**5**_**V**^**V**^**O**_**7**_^**1–**^,^43^**4-V**^**IV**^_**4**_**V**^**V**^_**2**_**O**_**7**_^**0**^,^43^**2-V**^**III**^**V**^**IV**^_**5**_**O**_**6**_^**1–**^,^65^ and **6-V**^**III**^**V**^**IV**^**V**^**V**^**O**_**6**_^**0**^(66) were synthesized according to literature precedent.

^1^H NMR spectra were recorded at 400 and 500 MHz on Bruker DPX-400 MHz and DPX-500 spectrometers, respectively, locked on the signal of deuterated solvents. All chemical shifts were reported relative to the peak of the residual hydrogen signal in deuterated solvents. MeCN-*d*_3_ was purchased from Cambridge Isotope Laboratories, degassed by three freeze–pump–thaw cycles, and stored over fully activated 3 Å molecular sieves. Electronic absorption measurements were recorded at room temperature in anhydrous MeCN in a sealed 1 cm quartz cuvette with an Agilent Cary 60 UV–vis spectrophotometer. Mass spectrometry analyses were performed on an Advion ExpressionL Compact mass spectrometer equipped with an electrospray probe and an ion-trap mass analyzer (instrument error: ±0.1 amu). Direct injection analysis was employed in all cases with a sample solution in MeCN.

Single crystals of [(C_4_H_9_)_4_N][(CH_3_)_2_NH_2_][V_6_O_7_(OCH_3_)_12_] were mounted on the tip of a thin glass optical fiber (goniometer head) and mounted on a XtaLab Synergy-S Dualflex diffractometer equipped with a HyPix-6000HE HPC area detector for data collection at 223 K. The structure was solved using *SHELXT-2018/2*(77) and refined using *SHELXL-2018/3*.^78^

#### Modified Synthesis of [^n^Bu_4_N][V_6_O_7_(OCH_3_)_12_] (**1-V**^**IV**^_**6**_**O**_**7**_^**2–**^)

A 48 mL thick-walled reaction vessel was charged with **4- V**^**IV**^_**4**_**V**^**V**^_**2**_**O**_**7**_^**0**^ (0.200 g, 0.253 mmol) and 20 mL of MeCN. A solution containing [^n^Bu_4_N][BH_4_] (0.195 g, 0.758 mmol, 3 equiv) in 10 mL of MeCN was added to the reaction mixture. The vessel was sealed with a Teflon cap, removed from the drybox, and stirred vigorously at 85 °C for 4 h, at which point the initial dark-green solution had changed to a vibrant sky blue. The vessel was returned to the drybox, and residual solvents were removed under reduced pressure. The resulting blue solid washed with tetrahydrofuran (3 × 15 mL) and filtered over a bed of Celite (2 cm) on a medium-porosity glass frit. The remaining blue solid was extracted in MeCN and dried *in vacuo* to yield **1-V**^**IV**^_**6**_**O**_**7**_^**2–**^ (0.378 g, 0.297 mmol, 92%).

#### Synthesis of [V_6_O_7_(OCH_3_)_12_(MeCN)][OTf] (**5-V**^**IV**^_**3**_**V**^**V**^_**3**_**O**_**7**_^**1+**^)

The synthesis of the oxidized cluster was adapted from a previous report.^43^ A 20 mL scintillation vial was charged with **4-V**^**IV**^_**4**_**V**^**V**^_**2**_**O**_**7**_^**0**^ (0.150 g, 0.190 mmol) and 5 mL of DCM. While stirring, a suspension of AgOTf (0.054 g, 0.21 mmol, 1.1 equiv) in DCM was added to the cluster-containing solution. The solution was stirred for 20 h, after which the formation of a gray precipitate was observed. The solution was filtered over a bed of Celite (2 cm) on a medium-porosity glass frit. The precipitate was washed with DCM (3 × 2 mL), and the resultant green solution was dried under reduced pressure to yield **5-V**^**IV**^_**3**_**V**^**V**^_**3**_**O**_**7**_^**1+**^ as a dark-green solid (0.174 g, 0.185 mmol, 98%).

#### Synthesis of [(C_4_H_9_)_4_N][(CH_3_)_2_NH_2_][V_6_O_7_(OCH_3_)_12_]

A 20 mL scintillation vial was charged with **1-V**^**IV**^_**6**_**O**_**7**_^**2–**^ (115.6 mg, 0.0907 mmol) and dissolved in 5 mL of MeCN. While stirring, a solution of (CH_3_)_2_NH_2_Cl (8.1 mg, 0.099 mmol) in MeCN was added dropwise, resulting in the immediate precipitation of a light-blue powder. The solid was washed with MeCN (3 × 5 mL) and extracted in methanol. Crystals suitable for analysis by X-ray diffraction were grown by vapor diffusion of Et_2_O into the methanol solution.

#### Acidification of Organic Bases

Acidification was performed using a preparation derived from literature precedent.^76^ A 10 mL solution of base in Et_2_O was charged in a 20 mL scintillation vial with a magnetic stirbar. The solution was vigorously stirred, and 0.95 equiv of HBF_4_·Et_2_O was added dropwise. ***Caution!** This reaction is exothermic. Take care to add HBF_4_·Et_2_O very slowly with stirring to avoid the solution boiling and splashing out of the vial.* Upon the addition of HBF_4_·Et_2_O to the stirring solution, a white solid immediately precipitated. The resultant powder was washed with Et_2_O (3 × 10 mL) and recrystallized from a mixture of 10:1 Et_2_O/MeCN. The recrystallized material was filtered over a medium-porosity frit, dried under reduced pressure overnight, and stored over P_2_O_5_ prior to use. The material purity was confirmed via ^1^H and ^13^C NMR spectroscopy. For previously reported acids, the referenced NMR data are noted in Table S1.^51,54,76,79−83^

Piperidinium tetrafluoroborate ([HPiperidine][BF_4_]). Yield: 82%. ^1^H NMR (500 MHz, CD_3_CN): δ 1.60 (m, 6H), 2.91 (t, 4H), 6.15 (bs, 2H). ^13^C NMR (128 MHz, CD_3_CN): δ 23.76, 25.06, 46.10.

*N*,*N*,*N*′,*N*′-Tetramethylpropylenediammonium tetrafluoroborate ([HTMPDA][BF_4_]). Yield: 75%. ^1^H NMR (500 MHz, CD_3_CN): δ 2.00 (quint, 2H), 2.73 (s, 12H), 3.04 (t, 4H), 7.38 (bs, 1H). ^13^C NMR (128 MHz, CD_3_CN): δ 20.50, 44.27, 57.33.

4-Chloro-2-nitroanilinium tetrafluoroborate ([4-Cl-2-NO_2_Aniline][BF_4_]). Yield: 84%. ^1^H NMR (500 MHz, CD_3_CN): δ 7.66 (d, 1H), 7.91 (dd, 1H), 8.38 (d, 1H), 8.71 (s, 3H). ^13^C NMR (128 MHz, CD_3_CN): δ 124.95, 127.65, 128.75, 136.63, 136.99, 142.88.

Triphenylammonium tetrafluoroborate ([HNPh_3_][BF_4_]). Yield: 31%. ^1^H NMR (500 MHz, CD_3_CN): δ 7.03 (m, 3H), 7.27 (t, 2H). ^13^C NMR (128 MHz, CD_3_CN): δ 123.88, 124.92, 130.31, 148.77.

## Supporting Information Available

^1^H NMR, electrospray ionization mass spectrometry, and electronic absorption spectra of POV clusters and acid addition reactions, charge-state calibration curves, and p*K*_a_ calculations for cluster charge states (PDF)

## Accession Codes

CCDC 2110688 contains the supplementary crystallographic data for this paper. These data can be obtained free of charge via www.ccdc.cam.ac.uk/data_request/cif, or by emailing data_request@ccdc.cam.ac.uk, or by contacting The Cambridge Crystallographic Data Centre, 12 Union Road, Cambridge CB2 1EZ, UK; fax: +44 1223 336033.
