# Supplementary material for: Charge-State Dependence of Proton Uptake in Polyoxovanadate-alkoxide Clusters
Source: Inorg Chem. 2022 Mar 16;61(12):4789–800. doi: 10.1021/acs.inorgchem.1c02937 (PMC8965876; doi:10.1021/acs.inorgchem.1c02937)
Supplement: Supplementary file 1 — ic1c02937_si_001.pdf [file ic1c02937_si_001.pdf]

Electronic Supporting Information File

Charge-state Dependence of Proton Uptake in Polyoxovanadate-alkoxide Clusters

Eric Schreiber, William W. Brennessel, and Ellen M. Matson*

Department of Chemistry, University of Rochester, Rochester, New York 14627, USA.

Corresponding author email: matson@chem.rochester.edu

Supporting Information Table of contents.

Methodology for Spectroscopic Determination of Cluster Basicity by Electronic Absorption and ^{1}H NMR Spectroscopies……S2
Figure S1. Crude ^{1}H NMR spectrum **1-$V^{IV}_6O_7^{2-}$** and $[HNEt_3][BF_4]$ in CD_3CN……S4
Figure S2. ESI-MS of crude reaction of **1-$V^{IV}_6O_7^{2-}$** and $[HNEt_3][BF_4]$……S4
Figure S3. ^{1}H NMR spectrum of a heated suspension of **1-$V^{IV}_6O_7^{2-}$** and $(Me)_2NH_2Cl$……S5
Table S1. List of acids used in this study with their pKas in acetonitrile……S6
Figure S4. ^{1}H-NMR spectra of **1-$V^{IV}_6O_7^{2-}$**, **2-$V^{III}V^{IV}_5O_6^{1-}$**, and **3-$V^{IV}_5V^{V}O_7^{1-}$**……S7
Figure S5. ^{1}H NMR spectra of **1-$V^{IV}_6O_7^{2-}$** and selected acids……S7
Figure S6. Electronic absorption spectra of **1-$V^{IV}_6O_7^{2-}$**, **2-$V^{III}V^{IV}_5O_6^{1-}$**, and **3-$V^{IV}_5V^{V}O_7^{1-}$** in CH_3CN at 21ºC……S8
Table S2. Electronic absorption parameters of **1-$V^{IV}_6O_7^{2-}$**, **2-$V^{III}V^{IV}_5O_6^{1-}$**, and **3-$V^{IV}_5V^{V}O_7^{1-}$** in CH_3CN at 21ºC, and calculations for the parameters of the spectrum of the conversion of **1-$V^{IV}_6O_7^{-2}$** by acidic protons……S8
Figure S7. Plot of the absorbance at 1000 nm for reactions between **1-$V^{IV}_6O_7^{2-}$** and acids in CH_3CN at 21ºC as a function of acid strength……S9
Figure S8. ^{1}H-NMR spectrum of **3-$V^{IV}_5V^{V}O_7^{1-}$** and $HNEt_3BF_4$ in CD_3CN……S9
Figure S9. ^{1}H-NMR spectra of **3-$V^{IV}_5V^{V}O_7^{1-}$**, **4-$V^{IV}_5V^{V}O_7^{0}$**, and **6-$V^{III}V^{IV}_5O_6^{0}$**……S10
Table S3. ^{1}H NMR parameters (chemical shift of $V_6O_7^n$ charge state mixture, relative integrations of paramagnetically shifted peaks) for reactions between **3-$V^{IV}_5V^{V}O_7^{1-}$** and organic acids recorded in CD_3CN at 21°C……S10
Figure S10. ^{1}H-NMR spectrum of oxidation of **6-$V^{III}V^{IV}_4V^{V}O_6^{0}$** with $AgBF_4$……S11
Figure S11. (a) ^{1}H-NMR spectra of mixtures of **4-$V^{IV}_4V^{V}_2O_7^{0}$** and **5-$V^{IV}_3V^{V}_3O_7^{+1}$** in CD_3CN at 21ºC, and (b) calibration curve of chemical shift……S11
References……S12

Methodology for Spectroscopic Determination of Cluster Basicity by Electronic Absorption and ^{1}H NMR Spectroscopies.

Determination of the Expected Molar Absorptivity for a 2:1:1 mixture of ***1-$V^{IV}{}_6O_7{}^{2-}$*****, *****3-$V^{IV}{}_5V^{V}O_7{}^{1-}$*****, and** ***2-$V^{III}V^{IV}{}_5O_6{}^{1-}$***. The reaction between **1-$V^{IV}{}_6O_7{}^{2-}$** and one equivalent of an acid which protonates one half the available clusters in solution should produce a 2:1:1 speciation of unreacted starting material and disproportionation products, respectively. This is based on the assumption that any and all clusters which take up a proton from the applied acid will react with another protonated cluster to produce equimolar amounts of **3-$V^{IV}{}_5V^{V}O_7{}^{1-}$** and **2-$V^{III}V^{IV}{}_5O_6{}^{1-}$**. Therefore, one half of the species in the reaction medium will be unreacted **1-$V^{IV}{}_6O_7{}^{2-}$**, and the remaining half will comprise the oxidized and O-atom vacant complexes. Using their molar absorptivities at 386 nm (see Table S2), the absorptivity at this wavelength can be calculated by multiplying by the known absorptivities of these species by their relative concentrations in solution and adding these values. For example, $\varepsilon_{386nm}{}^{1\text{-}V6O72\text{-}}$= 261 $M^{-1}cm^{-1}$, and its relative abundance in this theoretical mixture is 0.5; therefore, the contribution to the overall absorptivity of the reaction mixture would be 0.5*(261 $M^{-1}cm^{-1}$), or 130.5 $M^{-1}cm^{-1}$. The remaining species' contributions are listed in Table S2.

Data Fitting Details. Fitting of experimental data (both electronic absorption and ^{1}H NMR) was accomplished using the equation solver function in the data plotting software (Microsoft 365 Excel). The following general equation for a sigmoidal relationship was used for fitting:

$$\epsilon = A + \left(\frac{B-A}{1+\left(\frac{pKa}{C}\right)^{D}}\right)$$

Where A and B are the bounds of the equation, C is the midpoint of the sloped region, and D is a constant. A, B, C, and D were all solved for by the software by minimizing the sum of squared residuals versus the supplied raw data (molar absorptivity, chemical shift).

Determination of the Basicity of ***1-$V^{IV}{}_6O_7{}^{2-}$*** *using Electronic Absorption Spectroscopy.* To a stirred solution of **1-$V^{IV}{}_6O_7{}^{2-}$** of known concentration in MeCN was dropwise added 1 equivalent of an organic acid (for complete list of acids and pKa, see Table S1) from a stock solution in MeCN. The reaction was stirred for 3 h, then diluted. The electronic absorption spectrum of the crude reaction mixture was collected. The molar absorptivity of the IVCT band at 386 nm was plotted as a function of acid pKa. The resultant sigmoidal relationship was fitted using Microsoft Excel to determine the point at which 50% of **1-$V^{IV}{}_6O_7{}^{2-}$** was converted to **3-$V^{IV}{}_5V^{V}O_7{}^{1-}$** and **2-$V^{III}V^{IV}{}_5O_6{}^{1-}$**. The function was determined to be:

ε^{386nm} = 253.9518+(1868.1492-253.9518)/(1 +(**pKa**/19.2993)^639.4145)

The pKa was determined to be **19.28**.

Similarly, using the absorptivity of the band at 1000 nm (Table S2), a similar relationship was observed, producing the equation:

$$\varepsilon^{1000nm} = 146.9961+(376.7756-146.9961)/(1 +(\mathbf{pKa}/19.2815)^{\wedge} 700)$$

Using this wavelength, the pKa was determined to be **19.31**.

*^{1}H NMR Calibration Curve of Charge State Mixtures of **3-$V^{IV}_5V^VO_7^{1-}$** and **4-$V^{IV}_4V^V_2O_7^0$**.* Two solutions of **3-$V^{IV}_5V^VO_7^{1-}$** (0.01005 M) and **4-$V^{IV}_4V^V_2O_7^0$** (0.008051 M) in MeCN were combined in varying ratios (by volume) from 1:0 to 0:1, respectively to generate a total 1 mL solution. After shaking and allowing to sit for 5 min, the solutions were dried *in vacuo* and their ^{1}H NMR spectra were recorded in CD_3CN. The chemical shift of the resultant charge state mixture was plotted as a function of the fraction of **3-$V^{IV}_5V^VO_7^{1-}$** present in the mixture to produce the calibration curve (See manuscript Figure 4).

$\delta(V_6O_7^n)$ = 1.7229*(fraction of **1-$V_6O_7^{1-}$**) + 21.69 $R^2 = 0.9912$

*Determination of the basicity of **3-$V^{IV}_5V^VO_7^{1-}$** using ^{1}H NMR Spectroscopy.* To a stirred solution of **3-$V^{IV}_5V^VO_7^{1-}$** in 4 mL of MeCN was dropwise added 1 equivalent of an organic acid dissolved in 2 mL of MeCN. The reaction was stirred for 3 h, then dried *in vacuo*. ^{1}H NMR spectra of the dried reaction mixtures were recorded, and the chemical shift of the peak corresponding to the fully oxygenated POV-alkoxide $[V_6O_7(OCH_3)_{12}]^n$ (n = mixture of **3-$V^{IV}_5V^VO_7^{1-}$** and **4-$V^{IV}_4V^V_2O_7^0$** now present in solution) was recorded to determine the extent to which protonation of the cluster surface was accomplished. This was repeated with acids of pKa's ranging between 17.96 and 1.28. The chemical shift was plotted as a function of pKa and compared with the relevant calibration curve to determine the point at which 50% of **3-$V^{IV}_5V^VO_7^{1-}$** was converted to **4-$V^{IV}_4V^V_2O_7^0$** and **6-$V^{III}V^{IV}V^VO_6^0$** (See manuscript Figure 3). The pKa was determined to be **12.51**.

*^{1}H NMR Calibration Curve of Charge State Mixtures of **4-$V^{IV}_4V^V_2O_7^0$** and **5-$V^{IV}_3V^V_3O_7^{1+}$**.* An analogous procedure to determine the calibration curve between **3-$V^{IV}_5V^VO_7^{1-}$** and **4-$V^{IV}_4V^V_2O_7^0$** was performed using solutions of **4-$V^{IV}_4V^V_2O_7^0$** (0.01037 M) and **5-$V^{IV}_3V^V_3O_7^{1+}$** (0.01004 M). The resultant ^{1}H NMR shifts for the charge state mixtures were plotted as a function of the fraction of **2-$V_6O_7^0$** present in the mixture (Figure S11).

$\delta(V_6O_7^n)$ = 5.0905*(fraction of **1-$V_6O_7^0$**) + 16.84 $R^2 = 0.9944$

*Determination of the basicity of **4-$V^{IV}_4V^V_2O_7^0$** using ^{1}H NMR Spectroscopy.* An analogous procedure was followed for the determination of the pKa of the transient neutral hydroxylated cluster, $[V_6O_6(OH)]^0$, reacting **4-$V^{IV}_4V^V_2O_7^0$** with 1 equivalent of organic acid in MeCN. This was performed with acids of pKa's ranging between 9.1 and 1.28. The chemical shift was plotted as a function of pKa and compared with the relevant calibration curve to determine the point at which 50% of **2-$V_6O_7^0$** was

converted to **4-$V_6O_7^{1+}$** and the O-deficient [$V_6O_6(OCH_3)$]$^{1+}$ (See manuscript Figure 5). The pKa was determined to be **5.51**.

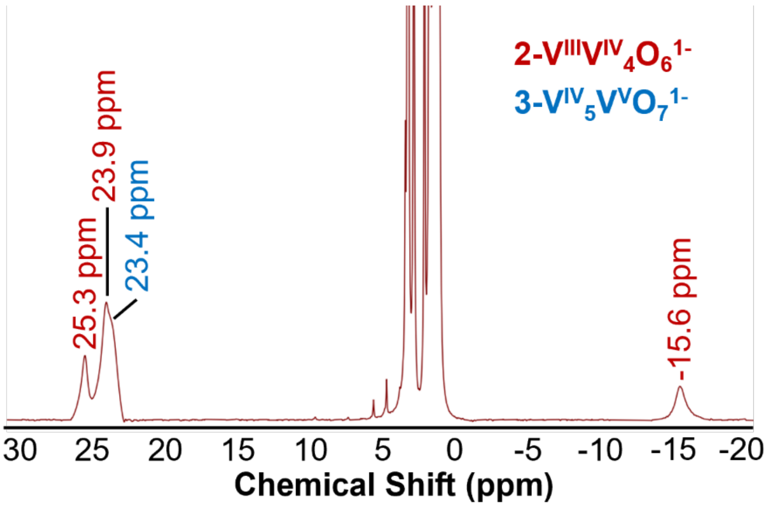

Figure S1. ^{1}H NMR spectrum of the reaction between **1-$V^{IV}_6O_7^{2-}$** and [$HNEt_3$][BF_4] in CD_3CN at 21°C.

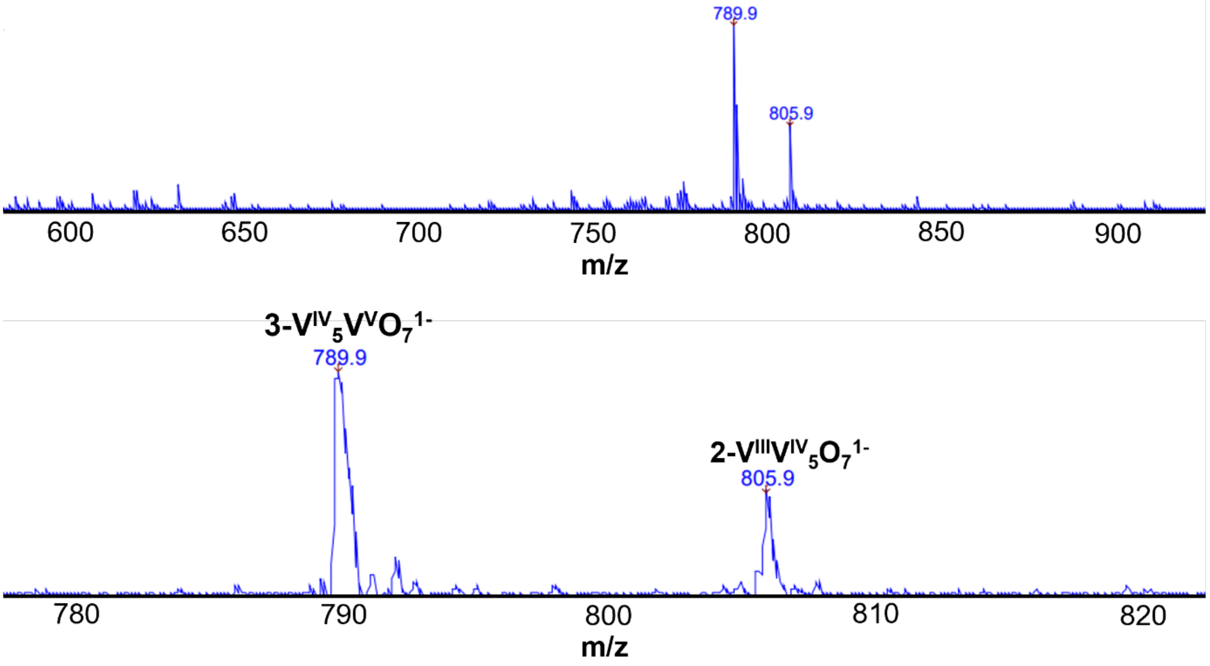

Figure S2. ESI-MS(-ve) of the reaction between **1-$V^{IV}_6O_7^{2-}$** and [$HNEt_3$][BF_4].

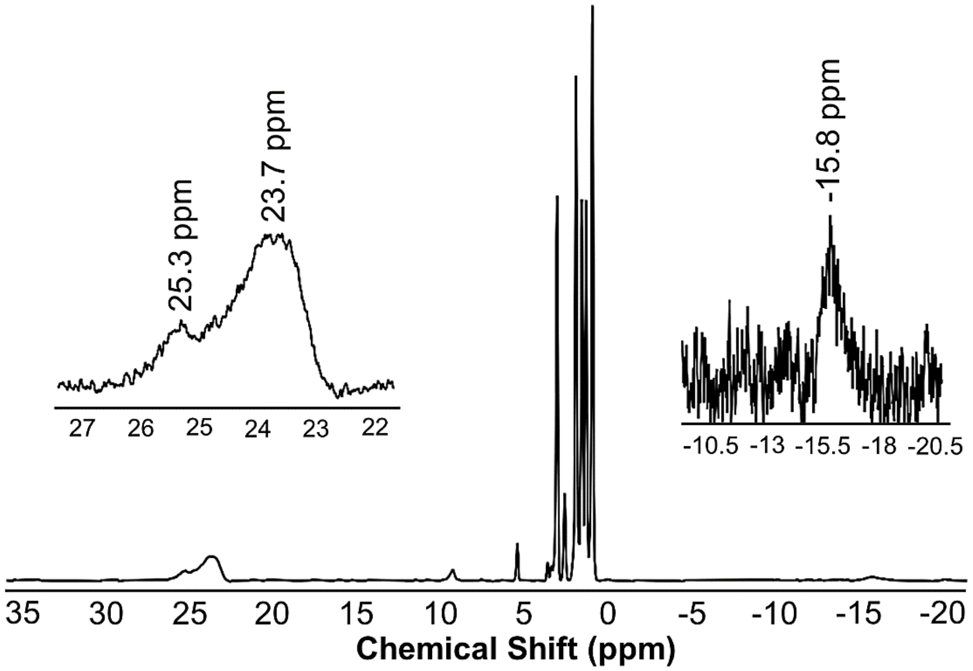

Figure S3. ^{1}H NMR spectrum of a suspension of **1-V$^{IV}_6$O$_7^{2-}$** and [(Me)$_2$NH$_2$]Cl heated to 50°C for 10 min (spectrum collected in CD_3CN at 21°C).

Table S1. List of acids used in this study with their pKas in acetonitrile.[1-13]

Acid	Abbreviation	pKa (MeCN)	pKa Reference	NMR Reference
Triphenylammonium BF_4	$HNPh_3BF_4$	1.28	17	*This work*
Tetrafluoroboric Acid Ether Complex	$HBF_4 \cdot Et_2O$	1.80	16	-
4-Chloro-2-Nitroanilinium BF_4	4-Cl-2-NO_2AnilineHBF_4	3.68	17	*This work*
2,6-Dichlorianilinium BF_4	2,6-DicloroanilineHBF_4	5.07	17	12
Diphenylammonium BF_4	$H_2Ph_2NBF_4$	5.98	17	10
2-Chloropyridinium BF_4	2-ClHPyrBF_4	6.79	17	12
2-Bromopyridinium BF_4	2-BrHPyrBF_4	7.02	18	12
3-Nitroanilinium BF_4	3-NO_2AnilineHBF_4	7.68	17	8
Pyrazolium BF_4	HPzBF_4	9.10	17	13
4-Bromoanilinium BF_4	4-BrAnilineHBF_4	9.44	17	6
N,N-Dimethylanilinium BF_4	N,N-DMAHBF_4	11.43	15	6
2-2’-Bipyridinium BF_4	2,2’-BipyHBF_4	12.27	17	7
Pyridinium BF_4	HPyrBF_4	12.53	17	11
2-Methylpyridinium BF_4	2-MeHPyrBF_4	13.28	17	11
4-MethoxyPyridinium BF_4	4-OMeHPyrBF_4	14.24	17	11
Trimethylammonium Chloride	$HNMe_3Cl$	17.61	14	-
4-Dimethylaminopyridinium BF_4	H(4-NMe_2)PyrBF_4	17.96	17	9
Triethylammonium BF_4	$HNEt_3BF_4$	18.82	17	11
Dimethylammonium Chloride	H_2NMe_2Cl	19.03	17	-
N,N,N’,N’-Tetramethyl-1,3-Propanediaminium BF_4	HTMPDABF_4	19.28	17	*This work*
Piperidinium BF_4	HPiperidineBF_4	19.35	17	*This work*
Pyrrolidinium BF_4	HPyrrolidineBF_4	19.62	17	11
Thiophenol	PhSH	22.6	10	-

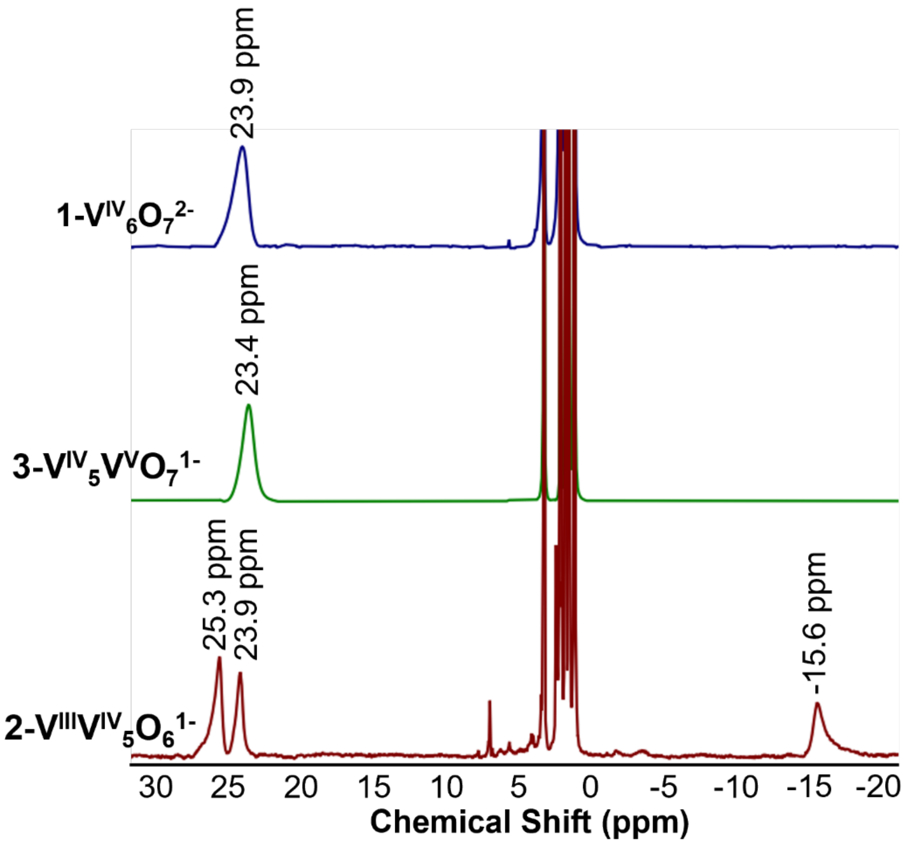

Figure S4. ^{1}H-NMR spectra of **1-V$^{IV}_6$O$_7$$^{2-}$**, **2-V^{III}V$^{IV}_5O_6$$^{1-}$**, and **3-V$^{IV}_5$V^{V}O$_7$$^{1-}$** in CD_3CN at 21ºC.

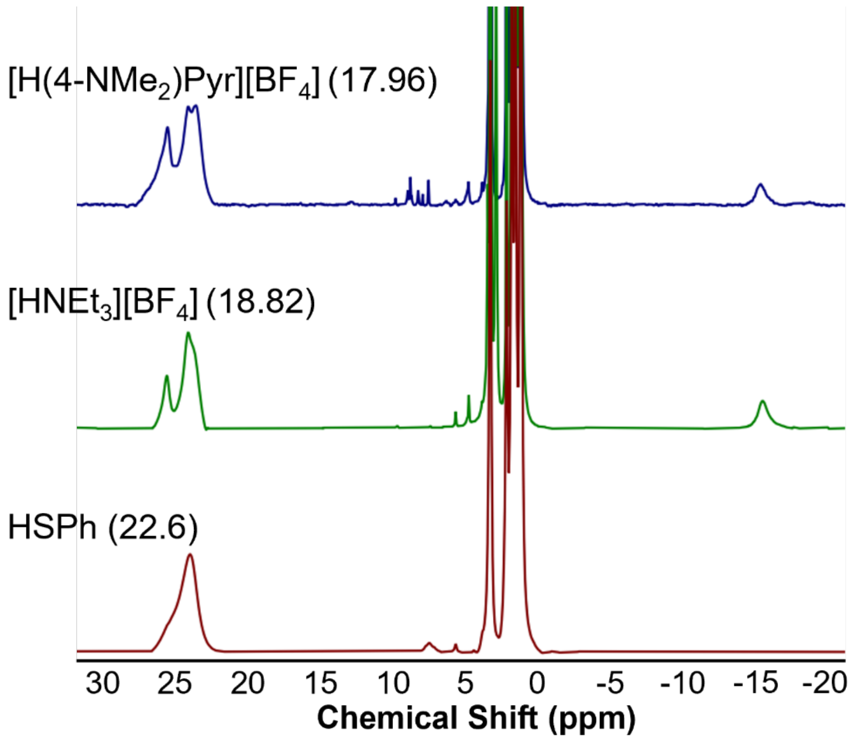

Figure S5. ^{1}H NMR spectrum of the reaction between **1-V$^{IV}_6$O$_7$$^{2-}$** and PhSH, $[HNEt_3][BF_4]$, and $[H(4\text{-}N(Me)_2Pyr][BF_4]$ in CD_3CN at 21°C.

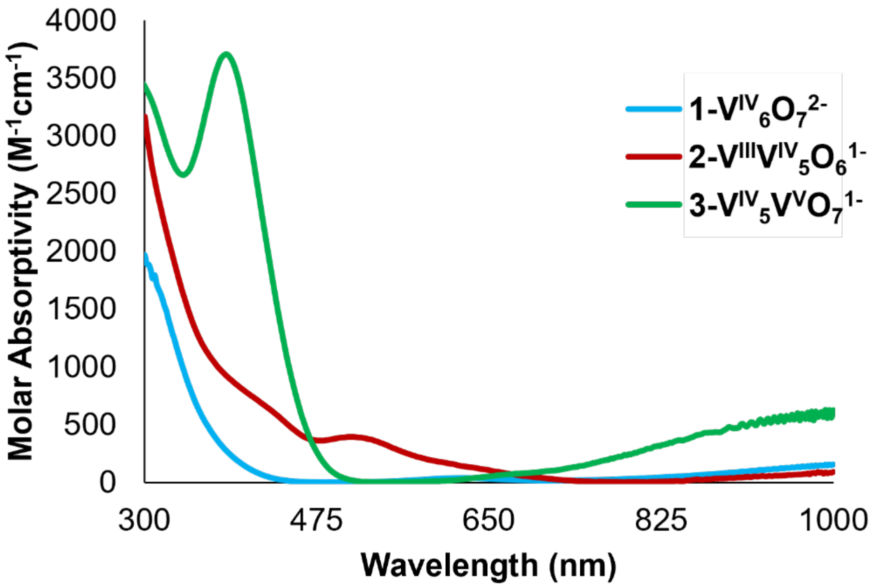

Figure S6. Electronic absorption spectra of **$1\text{-}V^{IV}_6O_7^{2-}$**, **$2\text{-}V^{III}V^{IV}_5O_6^{1-}$**, and **$3\text{-}V^{IV}_5V^{V}O_7^{1-}$** in CH_3CN at 21°C.

Table S2: Electronic absorption parameters of **$1\text{-}V^{IV}_6O_7^{2-}$**, **$2\text{-}V^{III}V^{IV}_5O_6^{1-}$**, and **$3\text{-}V^{IV}_5V^{V}O_7^{1-}$** in CH_3CN at 21°C, and calculations for the parameters of the spectrum of the conversion of **$1\text{-}V^{IV}_6O_7^{-2}$** by acidic protons.

**Complex**	ε_{386nm} **($M^{-1}cm^{-1}$)**	**Contribution to $\varepsilon_{386nm}^{rxn\ mixture}$ ($M^{-1}cm^{-1}$)**	ε_{1000nm} **($M^{-1}cm^{-1}$)**	**Contribution to $\varepsilon_{1000nm}^{rxn\ mixture}$ ($M^{-1}cm^{-1}$)**
**$1\text{-}V^{IV}_6O_7^{2-}$**	248	124	120	60
**$2\text{-}V^{III}V^{IV}_5O_6^{1-}$**	898	224.5	58	15.5
**$3\text{-}V^{IV}_5V^{V}O_7^{1-}$**	3694	923.5	525	131.25
	$\varepsilon_{386nm}^{rxn\ mixture}$:	**1272 $M^{-1}cm^{-1}$**	$\varepsilon_{1000nm}^{rxn\ mixture}$:	**204.75 $M^{-1}cm^{-1}$**

$$\varepsilon_{386\text{nm}}^{1-V_6^{IV}O_7^{2-},\ pKa} = \varepsilon_{386\text{nm}}^{1-V_6^{IV}O_7^{2-}} \times c\ (relative\ concentration\ at\ pKa\ condition)$$

$$\varepsilon_{386\text{nm}}^{1-V_6^{IV}O_7^{2-},\ pKa} = 248\ M^{1}\ cm^{-1} \times 0.5 = \mathbf{124\ M^{-1}\ cm^{-1}}$$

$$\varepsilon_{386\text{nm}}^{\text{rxn mixture}} = \varepsilon_{386nm}^{1-V_6^{IV}O_7^{2-},\ pka} + \varepsilon_{386nm}^{2-V^{III}V_5^{IV}O_6^{1-},\ pka} + \varepsilon_{386nm}^{3-V_5^{IV}V^{V}O_7^{1-},\ pka}$$

$$\varepsilon_{386nm}^{rxn\ mixture} = 124\ M^{-1}\ cm^{-1} + 224.5\ M^{-1}\ cm^{-1} + 923.5\ M^{-1}\ cm^{-1} = \mathbf{1272\ M^{-1}\ cm^{-1}}$$

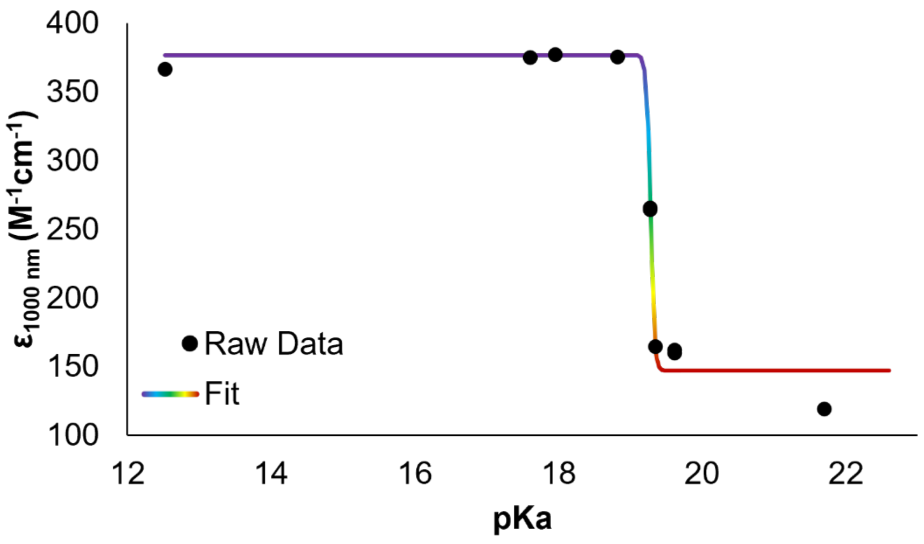

Figure S7. Plot of the absorbance at 1000 nm for reactions between **1-$V^{IV}{}_6O_7{}^{2-}$** and acids in CH_3CN at 21°C as a function of acidic strength.

Fit Line:

$$\in_{1000nm} = 147.00 + \frac{376.78 - 147.00}{1 + \left(\frac{pKa}{19.28}\right)^{700}}$$

$$pKa = \mathbf{19.31}$$

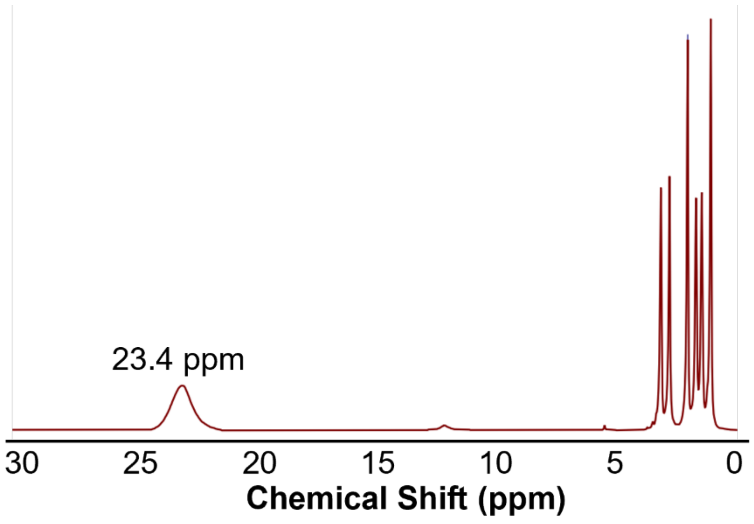

Figure S8. ^{1}H-NMR spectra of the reaction between **3-$V^{IV}{}_5V^{V}O_7{}^{1-}$** and $HNEt_3BF_4$ in CD_3CN at 21°C.

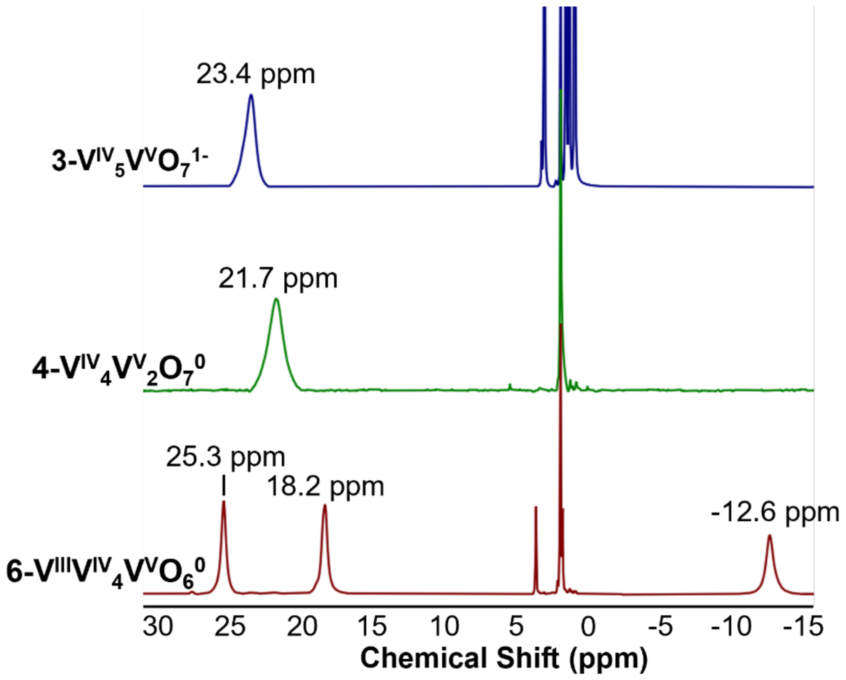

Figure S9. ^{1}H-NMR spectra of **3-$V^{IV}_5V^VO_7^{1-}$**, **4-$V^{IV}_5V^VO_7^0$**, and **6-$V^{III}V^{IV}_5O_6^0$** in CD_3CN at 21ºC.

Table S3. ^{1}H NMR parameters (chemical shift of $V_6O_7^n$ charge state mixture, relative integrations of paramagnetically shifted peaks) for reactions between **3-$V^{IV}_5V^VO_7^{1-}$** and organic acids recorded in CD_3CN.

Acid	pKa (MeCN)	$V_6O_7^n$ Chemical Shift (ppm)	Relative Integration (18.2 ppm)	Relative Integration (25.3 ppm + $V_6O_7^n$)
2-ClHPyrBF_4	6.79	21.66	1	3.93
2-BrHPyrBF_4	7.02	21.64	1	3.92
3-NO_2AnilineHBF_4	7.68	21.66	1	4.18
HPzBF_4	9.10	21.66	1	4.27
4-BrAnilineHBF_4	9.44	21.7	1	4.12
N,N-DMAHBF_4	11.43	21.82	1	4.64
2,2'-BipyHBF_4	12.27	22.08	1	4.79
HPyrBF_4	12.53	22.92	1	6.53
2-MeHPyrBF_4	13.28	23.26	1	16.89
4-OMeHPyrBF_4	14.24	23.34	1	23.94
H(4-NMe_2)PyrBF_4	17.96	23.38	n/a	n/a

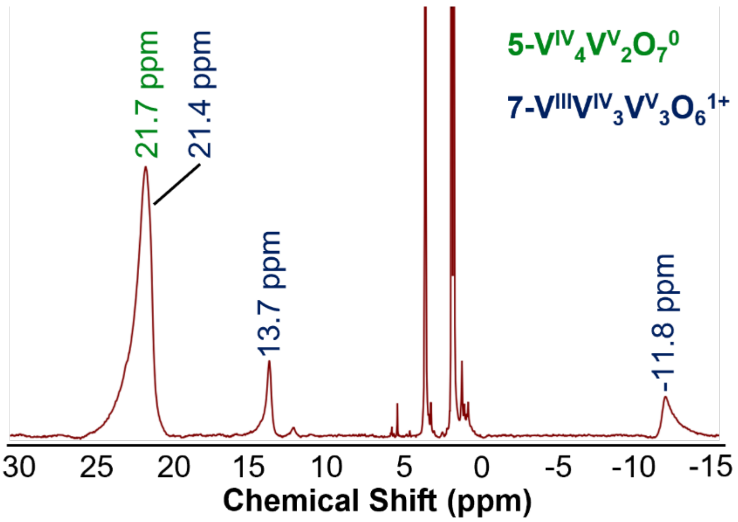

Figure S10. ^{1}H-NMR spectrum of the reaction mixture following oxidation of **6-$V^{III}V^{IV}{}_4V^{V}O_6{}^0$** by $AgBF_4$. Spectrum collected in CD_3CN at 21ºC.

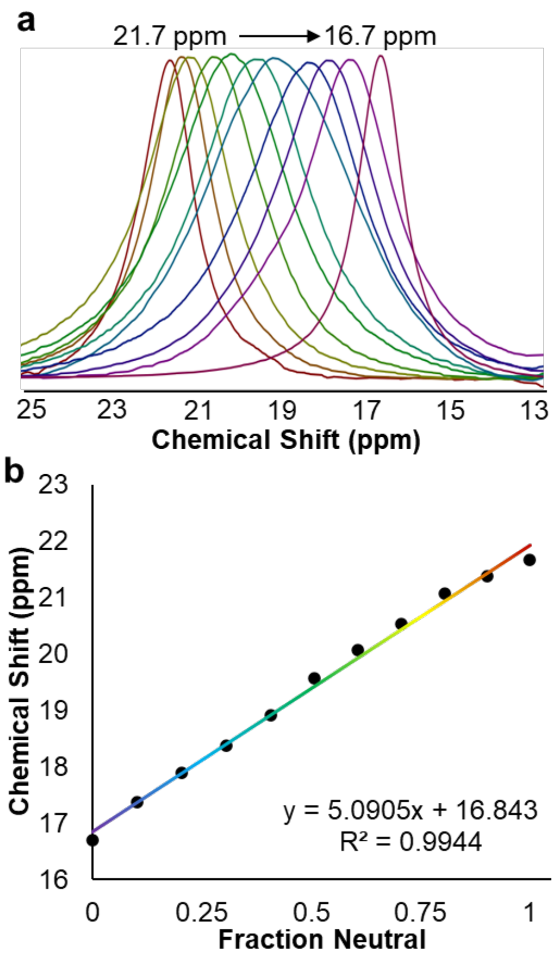

Figure S11. (a) ^{1}H-NMR spectra of charge state mixtures **of** ^{1}H-NMR spectra of **4-$V^{IV}{}_4V^{V}{}_2O_7{}^0$** and **5-$V^{IV}{}_3V^{V}{}_3O_7{}^{1+}$** in CD_3CN at 21ºC, and (b) NMR calibration curve of chemical shift.

References

1. Delpuech, J. J.; Bianchin, B.; Beguin, C., Proton transfer in methylammonium salts: a comparison of water and acetonitrile as solvents. *J. Chem. Soc., Chem. Commun.* **1970,** 1186-1187.

2. Milani, B.; Anzilutti, A.; Vicentini, L.; Sessanta O Santi, A.; Zangrando, E.; Geremia, S.; Mestroni, G., Bis-Chelated Palladium(II) Complexes with Nitrogen-Donor Chelating Ligands Are Efficient Catalyst Precursors for the CO/Styrene Copolymerization Reaction. *Organometallics* **1997,** *16*, 5064-5075.

3. Kaljurand, I.; Kütt, A.; Sooväli, L.; Rodima, T.; Mäemets, V.; Leito, I.; Koppel, I. A., Extension of the Self-Consistent Spectrophotometric Basicity Scale in Acetonitrile to a Full Span of 28 pKaUnits: Unification of Different Basicity Scales. *J. Org. Chem.* **2005,** *70*, 1019-1028.

4. KüTt, A.; Rodima, T.; Saame, J.; Raamat, E.; MäEmets, V.; Kaljurand, I.; Koppel, I. A.; Garlyauskayte, R. Y.; Yagupolskii, Y. L.; Yagupolskii, L. M.; Bernhardt, E.; Willner, H.; Leito, I., Equilibrium Acidities of Superacids. *J. Org. Chem.* **2011,** *76*, 391-395.

5. Pehlivan, L.; Métay, E.; Laval, S.; Dayoub, W.; Demonchaux, P.; Mignani, G.; Lemaire, M., Alternative method for the reduction of aromatic nitro to amine using TMDS-iron catalyst system. *Tetrahedron* **2011,** *67*, 1971-1976.

6. McCarthy, B. D.; Martin, D. J.; Rountree, E. S.; Ullman, A. C.; Dempsey, J. L., Electrochemical Reduction of Brønsted Acids by Glassy Carbon in Acetonitrile—Implications for Electrocatalytic Hydrogen Evolution. *Inorg. Chem.* **2014,** *53*, 8350-8361.

7. Thompson, E. J.; Berben, L. A., Electrocatalytic Hydrogen Production by an Aluminum(III) Complex: Ligand-Based Proton and Electron Transfer. *Angew. Chem. Int. Ed.* **2015,** *54*, 11642-11646.

8. Lathem, A. P.; Heiden, Z. M., Quantification of Lewis acid induced Brønsted acidity of protogenic Lewis bases. *Dalton Trans.* **2017,** *46*, 5976-5985.

9. McCarthy, B. D.; Dempsey, J. L., Decoding Proton-Coupled Electron Transfer with Potential–pKa Diagrams. *Inorg. Chem.* **2017,** *56*, 1225-1231.

10. Tshepelevitsh, S.; Kütt, A.; Lõkov, M.; Kaljurand, I.; Saame, J.; Heering, A.; Plieger, P. G.; Vianello, R.; Leito, I., On the Basicity of Organic Bases in Different Media. *Eur. J. Org. Chem.* **2019,** 6735-6748.

11. Guo, R.; Qi, X.; Xiang, H.; Geaneotes, P.; Wang, R.; Liu, P.; Wang, Y. M., Stereodivergent Alkyne Hydrofluorination Using Protic Tetrafluoroborates as Tunable Reagents. *Angew. Chem. Int. Ed.* **2020,** *59*, 16651-16660.

12. Matuszek, K.; Vijayaraghavan, R.; Forsyth, C. M.; Mahadevan, S.; Kar, M.; Macfarlane, D. R., Pyrazolium Phase-Change Materials for Solar-Thermal Energy Storage. *ChemSusChem* **2020,** *13*, 159-164.

13. Mech, P.; Bogunia, M.; Nowacki, A.; Makowski, M., Calculations of pKaValues of Selected Pyridinium and Its N-Oxide Ions in Water and Acetonitrile. *J. Phys. Chem. A* **2020,** *124*, 538-551.
